# The members of the miR-148/152 family inhibit cancer stem cell-like properties in gastric cancer via negative regulation of ITGA5

**DOI:** 10.1186/s12967-023-03894-1

**Published:** 2023-02-10

**Authors:** Xiaoying Li, Lin Li, Jiangying Wu

**Affiliations:** 1 Department of Oncology, The Fourth Affiliated Hospital of China Medical University, Shenyang, 110032 People’s Republic of China; 2 Department of Intervention, The Fourth Affiliated Hospital of China Medical University, No. 4, Chongshan East Road, Huanggu District, Shenyang, 110032 Liaoning People’s Republic of China

**Keywords:** Gastric cancer, Cancer stem cell-like properties, MicroRNA-148/152 family, ITGA5

## Abstract

**Background:**

The role of microRNA (miRNA) in modulating the function of cancer stem cells through diverse signaling pathway has been evidenced. We here identified a role of microRNA (miRNA) family, specifically miR-148/152, in gastric cancer and delineated its functional effects on gastric cancer stem cells.

**Methods:**

Bioinformatics analysis was conducted to analyze expression of integrin α5 (ITGA5) which was verified through expression determination in clinical tissue samples. Next, the upstream regulatory factors of ITGA5 were determined. CD44^+^EpCAM (high) cells sorted from AGS cells subjected to gain-of-function experiments, followed by evaluation of their capacity of colony formation, generation of tumorosphere, cell migration and viability in vitro and xenograft tumor formation in vivo.

**Results:**

ITGA5 was elevated in gastric cancer tissues and confirmed as a target gene of the miR-148/152 family members. The miR-148/152 family members were downregulated in gastric cancer tissues and cells. Decreased expression of miR-148/152 family members was also detected in gastric cancer stem cells. However, the raised expression led to reduced colony formation, tumorosphere, cell migration, cell viability, and drug resistance of CD44+EpCAM (high) AGS cells in vitro, and tumorigenesis in vitro. ITGA5 overexpression reversed the effect of the miR-148/152 family members.

**Conclusions:**

This study demonstrates that the miR-148/152 family members may prevent gastric cancer stem cell-like properties by targeting ITGA5, which can serve as an appealing target for gastric cancer treatment.

**Supplementary Information:**

The online version contains supplementary material available at 10.1186/s12967-023-03894-1.

## Background

Gastric cancer is one of the most common malignant types of tumors, with more than one million annually diagnosed cases worldwide [1]. Despite marked improvements in diagnosis and prevention, gastric cancer is still ranked sixth in incidence and second in mortality across 185 countries in 2018 [2]. Surgical procedures such as laparoscopy-assisted distal gastrectomy and open distal gastrectomy are considered standard treatment options for gastric cancer at the early stage [3]. Unfortunately, gastric cancer is usually asymptomatic in its early stages and is typically diagnosed when many patients are at the advanced stage, which renders the tumor inoperable at the time of diagnosis [4]. Cancer stem cells (also called tumor-initiating cells) are enriched in side population (SP) cells and have the capability to maintain self-renewal and differentiation properties, thus facilitating tumor growth and enhancing metastatic potential [5]. Recent research supports the presence of cancer stem cells in solid tumors of various organs, including gastric cancer [6]. The expanding research field of gastric cancer stem cell biology includes the characterization of candidate biomarkers with potential diagnostic and therapeutic implications. However, specific molecular mechanisms underlying cancer stem cell features remain under-studied.

Integrin α5 (ITGA5), a member of the integrin adhesion molecule family, has been implicated in the metastasis and oncogenesis of cancer [7, 8]. For instance, the expression of ITGA5 is elevated in colorectal cancer tissues and cells and this elevation can enhance colorectal cancer cell growth and tumorigenesis while decreasing cell apoptosis [9]. More importantly, ITGA5 has been characterized to be upregulated in gastric cancer, and its high expression indicates the poorer survival of patients with gastric cancer [10]. Suppression of ITGA5 can significantly inhibit stem-cell like properties in hepatocellular carcinoma [11]. Nonetheless, the possible effect of ITGA5 on the gastric cancer stem-cell like properties remains elusive.

TargetScan database used in this study predicted ITGA5 as a putative target gene of the microRNA (miR)-148/152 family members. Accruing evidence over the past few years indicate miRNAs as critical modulators of cancer stem cell generation and the maintenance of cancer stem cell characteristics. miRNAs are RNA transcripts that are typically in the length of 18 to 24 nucleotides. They may function as either tumor suppressors or oncogenes, by binding to their target mRNAs and thus regulating gastric carcinogenesis [12]. The miR-148/152 family consisting of miR-148a, miR-148b, and miR-152 is differentially expressed in gastric cancer tissues as compared to that of tumor-free gastric tissues, and is also shown to modulate the initiation of gastric cancer [13, 14]. In our previous study, bioinformatics analysis showed that the expression of ITGA5 was significantly increased in gastric cancer, and miR-148/152 family members all had targeted binding sites with ITGA5. Therefore, we suspect that miR-148/152 family may have low expression in gastric cancer, while overexpression of miR-148/152 family members could target and inhibit the ITGA5 gene, thereby inhibiting the self-renewal ability, clonal formation ability and drug resistance of gastric cancer stem cells, and ultimately preventing the phenotypic formation of gastric cancer stem cells.

## Materials and methods

### Ethics statement

The study protocol was ratified by the Ethics Committee of the Fourth Affiliated Hospital of China Medical University and all procedures were compliant with the *Declaration of Helsinki*. All of the included subjects have submitted written informed consents. Animal experiments were approved by the Animal Ethics Committee of the Fourth Affiliated Hospital of China Medical University and performed according to the Guide for the Care and Use of Laboratory animals published by the US National Institutes of Health.

### Gastric cancer-related gene expression datasets and differential gene screening

Gastric cancer-related gene expression datasets were retrieved from the Gene Expression Omnibus (GEO) database with “gastric cancer” as the key word. The R “inSilicoMerging” software package was used to merge multiple gastric cancer-related gene expression datasets [15]. The batch effect was removed using the previously described method [16] to obtain the matrix following batch effect elimination. Differential analysis of the gene expression datasets was then conducted using the R “limma” package (version 3.50.0) [17] with |logFoldChange| > 1 and adjusted *p* value < 0.05 as the threshold to screen the differentially expressed genes (DEGs).

The known genes related to gastric cancer were retrieved from the MalaCards database [18]. A gene interaction network was made employing the STRING database. miRNAs regulated by ITGA5 were predicted by the DIANA-microT-CDS [19], miRDB [20], mirDIP [21] and TargetScan (version 7.1) [22] databases. The top 30 miRNAs from the conserved entry sites within the prediction results from the TargetScan database (sorted by conserved branch length score) were selected for Venn diagram analysis. The used R software version is 4.0.5 and R Studio software version is 1.3.

### Collection of clinical samples

Cancer and adjacent normal tissues (5–10 cm away from the cancer tissues) were surgically obtained from 52 patients with gastric cancer (42 males and 10 females; 32–68 years with a mean age of 48 years) admitted into Gastrointestinal Surgery of the Fourth Affiliated Hospital of China Medical University from January 2013 to January 2014. Inclusion and exclusion criteria were conducted as previously described [23]. On the basis of the American Joint Committee on Cancer tumor-node-metastasis cancer staging system, 39 cases were at stage I–II, and 13 cases at stage III–IV; 36 cases with the tumor size < 5 cm and 16 cases with the tumor size ≥ 5 cm; 40 cases with well-moderate differentiation and 12 cases with poor differentiation; 24 cases without lymph node metastasis and 28 cases with lymph node metastasis; 47 cases without distant metastasis, and 5 cases with distant metastasis [24, 25]. The detailed information is shown in Additional file 2: Table S1. The samples were immediately frozen in liquid nitrogen after rinsed with normal saline and used for subsequent analysis. The follow-up duration ranged from 3 to 60 months, with a median follow-up of 60 months.

### Cell culture

Human gastric cancer cell lines MKN45, AGS, KATO-III, NCI-N87, and SNU-1, and normal immortalized gastric mucosal cells (GES-1) were grown in the Roswell Park Memorial Institute 1640 medium (Gibco, Carlsbad, CA) appended to 10% fetal bovine serum (FBS; Hangzhou Sijichun Co., Ltd., Hangzhou, Zhejiang, China), 100 U/mL penicillin and 100 mg/mL streptomycin (Gibco) in a 5% CO_2_ incubator at 37 °C. GES-1 and MKN45 cells were procured from Shanghai Zhong Qiao Xin Zhou Biotechnology Co., Ltd., (Shanghai, China) and the remaining cells from Shanghai Institute of Cell Biology, Chinese Academy of Sciences (Shanghai, China).

### Luciferase assay

Artificially synthesized wild type ITGA5 (ITGA5-Wt) and ITGA5-mutant (ITGA5-Mut) were transfected into HEK293T cells in the presence of the miR-148/152 mimic. Luciferase activity was assayed employing a dual-luciferase reporter assay system kit (RG005, Beyotime Biotechnology Co., Ltd., Shanghai, China) [26].

### Cell sorting by flow cytometry and transient transfection

CD44^+^EpCAM (high) SP cells were isolated from AGS cells by flow cytometric cell sorting, as previously described by Gao et al. [27]. SP and non-SP cells were assayed for the generation of tumorosphere and the detection of stemness-related gene expression profiles (CD133, CD44, OCT-4, MDR1, EpCAM, ABCG2, and CD24). The plasmids (Ruibo, Guangzhou, Guangdong, China) of miR-148a mimic, miR-148b mimic, miR-152 mimic, sh-ITGA5, and oe-ITGA5 were introduced into CD44^+^EpCAM (high) cells with the help of the Lipofectamine 3000 reagent (L3000001, Thermo Fisher, Waltham, MA). The concentration of the used plasmids was 80 nM. After transfection for 6 h, the cells continued to culture for 48 h with new medium and collected for subsequent experimentations.

### Colony formation assay

A single cell suspension of CD44^+^EpCAM (high) cells was grown with 10% FBS-supplemented Dulbecco’s modified Eagle’s medium (DMEM) (renewed every 4 days) in a 6-well plate (1 × 10^3^ cells/well). Two weeks later, colonies were subjected to 4% paraformaldehyde fixation and 0.1% crystal violet staining (Sigma). Afterwards, photographs were taken and the number of clones was counted using ImageJ software. The number of clones > 50 cells was counted employing a microscope at a low magnification [28].

### Sphere-forming assay

CD44^+^EpCAM (high) cells were seeded in a commercially available 24-well ultra-low-attachment plate at 1000 cells per well and left to grow in serum-free DMEM-F12 for 5 days. A Nikon EclipseTE2000-S microscope was used to observe the generation of tumorospheres and the number of tumorospheres was calculated: sphere-forming rate (%) = the average number of tumor spheres in each well/the number of cells seeded in each well (1 × 10^3^) × 100% [29].

### Transwell assay

CD44^+^EpCAM (high) cells were prepared into a suspension of 1 × 10^5^ cells/μL using 100 μL serum-free DMEM and were then added to the upper chambers. After cells were incubated for 12 h at 37 °C, they were later transferred to the lower chambers containing 10% FBS-supplemented DMEM and were fixed for 30 min with 100% paraformaldehyde and stained for 15 min with 0.1% crystal violet. Stained cells were counted in five random microscopic fields per well [30].

### CCK-8 assay

Cell viability was examined in a commercially available CCK-8 kit (96992, Sigma, Shanghai, China). CD44^+^EpCAM (high) cells were cultured for 0, 1, 2, 3, and 4 days and 10 µL CCK-8 solution was added to each well at the end of cell culture and was left to incubate for an additional 4 h [31]. In addition, CD44^+^EpCAM (high) cells were incubated with different concentrations of 5-fluorouracil (5-FU) (2, 4, 6, and 8 nM) for 48 h, followed by examination of cell viability [32].

### In vivo tumor formation by CD44^+^EpCAM (high) cells

Totally, 48 specific pathogen-free male BALB/c nude mice (aged: 3–5 weeks; weighing: 18–20 g; Experimental Animal Center of Sun Yat-sen University) were housed at a constant temperature of 25–27 °C with humidity of 45–50%. The mice were then subcutaneously injected with 0.1 mL resuspension containing 1 × 10^6^ cells stably transfected with mimic-NC, miR-148a mimic, miR-148b mimic and/or miR-152 mimic into the right side of the back (n = 8 mice for each treatment). After inoculation, the length and width of the tumor in mice were measured using digital calipers every 4 days, followed by calculation of the tumor volume and construction of a growth curve. All mice were euthanized 25 days after inoculation, and the tumor was removed and weighed [33].

### Immunohistochemical staining

Tumor sections from nude mice with CD44^+^EpCAM (high) cell xenografts were deparaffined, hydrated, blocked, and incubated with rabbit polyclonal antibodies to ITGA5 (1: 100, ab150361, Abcam, Cambridge, UK) at 4 °C overnight, followed by incubation with goat anti-mouse IgG (1: 1000, ab6785, Abcam) for 30 min. Five fields of view at 200× magnification were randomly selected for visualization for each replicate using an inverted microscope (CX41-12C02, Olympus, Tokyo, Japan) [34].

### RT-qPCR

Extracted tissue and cell RNA contents by TRIzol reagents (Takara, Dalian, Liaoning, China) were used to generate cDNA employing the PrimeScript RT reagent Kit (RR047A, Takara). Quantification of miRNA and mRNA expression was performed using SYBR green-based RT-qPCR. Primer information is listed in Additional file 3: Table S2. The expression of miR-148a, miR-148b and miR-152 was normalized to that of U6 while the expression of the remaining genes to GAPDH. The fold changes were calculated employing the 2^−△△CT^ method [35].

### Western blot

Total protein was extracted from cells using PMSF-containing RIPA lysis buffer (R0010, Solarbio, Beijing, China) and then separated with 10% SDS-PAGE and then wet-transferred onto the membrane. The membrane was probed with primary rabbit antibodies to ITGA5 (1: 1000, ab150361, Abcam) and GAPDH (1: 2500, ab9485, Abcam) and re-probed with horseradish peroxidase-labeled secondary antibody goat anti-rabbit IgG. Visualization of immunoreactive bands was performed with the help of enhanced chemiluminescence reagents (BB-3501, Amersham Pharmacia, Little Chalfont, UK) [35].

### Statistical analysis

All data (mean ± standard deviation) were suggestive of three independent experiments (each in triplicate). Comparisons of data between two groups with normal distribution and homogeneity of variance were made using independent sample *t*-test while the comparison between gastric cancer tissues and adjacent normal tissues was done using paired *t*-test. Data comparisons between multiple groups were completed employing one-way analysis of variance (ANOVA) with Tukey’s post hoc test. Tumor volume at different time points was assayed by repeated measures of ANOVA and optical density values at different time points were assayed by Bonferroni-corrected two-way ANOVA. Pearson’s correlation coefficient was adopted for evaluation of the correlation between two indicators. Survival curves were constructed using Kaplan–Meier’s method, and statistical differences were evidenced by a log-rank test. All statistical analyses were processed utilizing SPSS 21.0 software (IBM, Armonk, NY), with *p* < 0.05 deemed as statistical significance.

## Results

### Bioinformatics prediction reveals that miR-148/152 family members may be involved in the occurrence and development of gastric cancer by targeting ITGA5

**Fig. 1:**
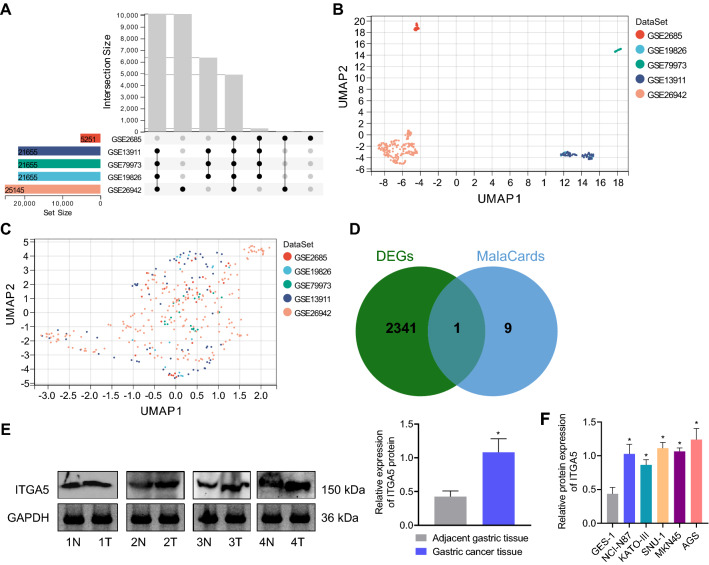
Bioinformatics prediction of gastric cancer-related miRNAs and genes. **A** A UpSet plot of the merged GSE2685, GSE13911, GSE19826 and GSE26942 gene expression datasets. The left histogram shows the total amount of elements contained in each original gene expression dataset, the below intersection dot refers to the corresponding datasets on the left, and the connection between the dots indicates the presence of intersection between the corresponding datasets. **B** UMAP diagram before batch effect removal. **C** UMAP diagram after batch effect removal. **D** Venn diagram of the DEGs in gastric cancer tissue samples from GSE2685, GSE13911, GSE19826 and GSE26942 gene expression datasets and the known genes related to gastric cancer from the MalaCards database. **E** Detection of ITGA5 expression in gastric cancer and adjacent normal tissue by Western blot (N means adjacent normal tissue, T means gastric cancer tissue, sample number is 4). **F** The expression of miR-148a, miR-148b, and miR-152 in gastric cancer cell lines (MKN45, AGS, KATO-III, NCI-N87, and SNU-1) and normal immortalized gastric mucosal cells GES-1 examined by RT-qPCR. *p < 0.05 compared with adjacent normal tissue samples or GES-1 cells

We downloaded gastric cancer-related gene expression datasets GSE2685, GSE13911, GSE19826, GSE26942, and GSE79973 from the GEO database and merged the expression data of these gene expression datasets (Fig. 1A) to determine the DEGs in gastric cancer samples. UMAP graph described that the samples between each dataset had batch effects (Fig. 1B). After removing the batch effect, the results of the UMAP graph revealed that the samples among the datasets were clustered and intertwined with each other (Fig. 1C), indicating the better removal of the batch effect. The results of differential analysis of the merged gene expression datasets using the R “limma” package revealed 2342 DEGs (Additional file 4: Table S3). These DEGs were then intersected with the top 10 known genes related to gastric cancer obtained from the MalaCards database, with ITGA5 identified (Fig. 1D).

Following Western blot analysis, we found a high level of ITGA5 in gastric cancer tissues (Fig. 1E). Moreover, similar high expression of ITGA5 was detected in five gastric cancer cells relative to GES-1 cell, among which AGS cells showed the highest ITGA5 expression; thus AGS cells were selected for further experiments (Fig. 1F).

### ITGA5 was highly expressed in CD44^+^EpCAM^+^ cells

**Fig. 2:**
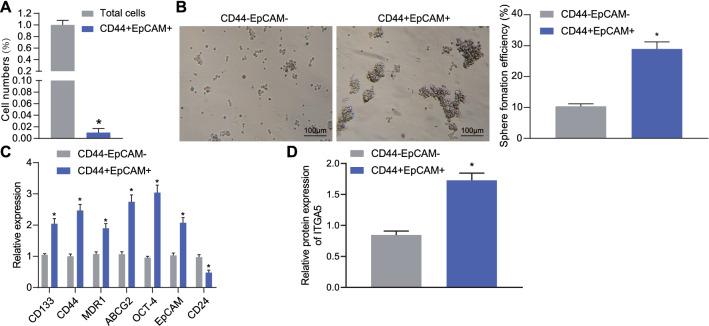
ITGA5 is highly expressed in CD44^+^EpCAM^+^ cells. **A** The proportion of CD44^+^EpCAM^+^ cells by SP sorting using flow cytometric analysis. **B** Sphere-forming ability of CD44^−^EpCAM^−^ cells and CD44^+^EpCAM^+^ cells examined by sphere-forming assay (scale bar: 100 μm). **C** The expression of CD133, CD44, OCT-4, MDR1, EpCAM, ABCG2, and CD24 in CD44^−^EpCAM^−^ cells and CD44^+^EpCAM^+^ cells examined by RT-qPCR. **D** Expression of ITGA5 in CD44^−^EpCAM^−^ cells and CD44^+^EpCAM^+^ cells examined by RT-qPCR. *p < 0.05 compared with CD44-EpCAM^−^ cells. Measurement data are expressed as mean ± standard deviation. Comparisons between two groups were analyzed using unpaired *t*-test. The cell experiment was run in triplicate independently

In order to explore the expression of ITGA5 in gastric can cer stem cells, SP sorting by flow cytometric analysis showed that in AGS cell lines, CD44^+^EpCAM^+^ cells accounted for 1.0% (Fig. 2A). CD44^+^EpCAM^+^ cells and CD44^−^EpCAM^−^ cells were sorted out by flow cytometry, and the sphere-forming assay showed that CD44^+^EpCAM^+^ cells had stronger sphere-forming ability than CD44^−^EpCAM^−^ cells (Fig. 2B). Determination utilizing RT-qPCR revealed that compared with CD44^−^EpCAM^−^ cells, the expression of CD133, CD44, OCT-4, MDR1, EpCAM and ABCG2 in CD44^+^EpCAM^+^ cells was significantly increased, while the expression of CD24 was significantly decreased (Fig. 2C). In addition, we found that the expression of ITGA5 in CD44^+^EpCAM^+^ cells was lower than that in CD44^−^EpCAM^−^ cells (Fig. 2D). Therefore, CD44^+^EpCAM^+^ cells were selected as gastric cancer stem cells for subsequent experiments.

### Silencing ITGA5 inhibited the stem cell properties of CD44^+^EpCAM^+^ cells

**Fig. 3:**
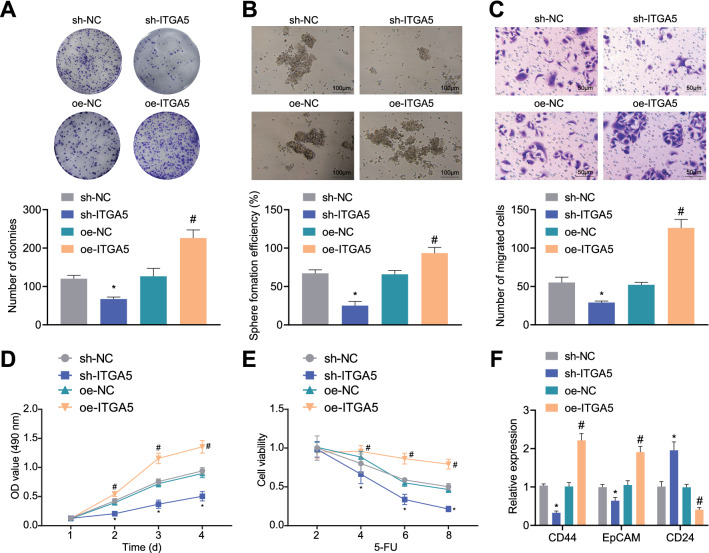
Silencing ITGA5 inhibits the stem cell properties of CD44^+^EpCAM^+^ cells. **A** Numbers of colonies of CD44^+^EpCAM^+^ cells following overexpression of ITGA5. **B** Sphere-forming ability of CD44^+^EpCAM^+^ cells examined by sphere-forming assay. **C** Numbers of migrated CD44^+^EpCAM^+^ cells following overexpression of ITGA5. **D** Viability of CD44^+^EpCAM^+^ cells following overexpression of ITGA5 measured by CCK-8 assay. **E** Drug resistance of CD44^+^EpCAM^+^ cells following overexpression of ITGA5 measured by CCK-8 assay. **F** Expression of CD44, EpCAM and CD24 in CD44^+^EpCAM^+^ cells following overexpression of ITGA5 measured by RT-qPCR. *p < 0.05 compared with sh-NC. ^#^*p* < 0.05 compared with oe-NC

We then explore the effect of ITGA5 on gastric cancer stem cells. We found that the colony formation and sphere-forming abilities were reduced following sh-ITGA5 treatment, while oe-ITGA5 treatment led to opposite trends (Fig. 3A, B). Besides, we also identified that cell migration, viability and drug resistance were inhibited upon sh-ITGA5 treatment, while oe-ITGA5 treatment led to opposite trends (Fig. 3C–E). Further expression determination using RT-qPCR revealed that expression of CD44 and EpCAM was reduced but CD24 expression was increased upon sh-ITGA5 treatment, while oe-ITGA5 treatment led to opposite trends (Fig. 3F). Collectively, silencing of ITGA5 suppressed the stem cell properties of CD44^+^EpCAM^+^ cells.

### The miR-148/152 family genes may be involved in the progression of gastric cancer by targeting ITGA5 regulation

**Fig. 4:**
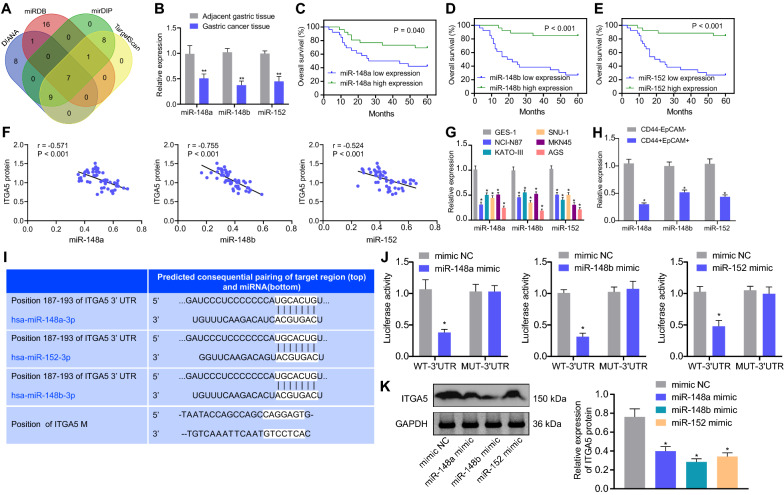
The miR-148/152 family genes may be involved in the progression of gastric cancer by targeting ITGA5. **A** Prediction of miRNA for ITGA5 regulation. The four ovals in the figure represent the prediction results of ITGA5-regulated miRNA in the four databases respectively, and the middle part represents the intersection of the prediction results of the four databases. **B** The expression of miR-148a, miR-148b, and miR-152 in 52 pairs of gastric cancer and adjacent normal tissue samples (n = 52) examined by RT-qPCR. *p < 0.05 and **p < 0.01 compared with adjacent normal tissue samples. **C** Kaplan–Meier curve analysis of the correlation of the overall survival of 52 patients with gastric cancer with miR-148a expression. **D** Kaplan–Meier curve analysis of the correlation of the overall survival of 52 gastric cancer patients with miR-148b expression. **E** Kaplan–Meier curve analysis of the correlation of the overall survival of 52 patients with gastric cancer with miR-152 expression. **F** Pearson analyzed the correlation between miR-148/152 family genes and ITAG5 genes in clinical cancer samples. **G** The expression of miR-148a, miR-148b, and miR-152 in gastric cancer cell lines (MKN45, AGS, KATO-III, NCI-N87, and SNU-1) and normal immortalized gastric mucosal cells GES-1 examined by RT-qPCR. *p < 0.05 compared with GES-1 cells. **H** The expression of miR-148/152 family genes in CD44^+^EpCAM^+^ cells and CD44^−^EpCAM^−^ was detected by RT-qPCR. **I** TargetScan software predicted the potential target-binding sites of miR-148/152 and ITGA5. **J** Binding of miR-148a, miR-148b, and miR-152 to ITGA5 confirmed by dual-luciferase reporter assay. **K** Western blots and quantification of ITGA5 protein in AGS cells treated with miR-148a mimic, miR-148b mimic, and miR-152 mimic, normalized to GAPDH. *p < 0.05 compared with GSE-1 cells, CD44^−^EpCAM^−^ or mimic-NC. The cell experiment was run in triplicate independently

To further understand the upstream regulatory mechanism of ITGA5 in gastric cancer, we first used bioinformatics databases such as DIANA to predict the upstream miRNA regulated by ITGA5. Then, the intersection of the prediction results of the four databases was taken (Fig. 4A). Finally, we obtained seven potential upstream miRNAs regulated by ITGA5 genes. Among the seven miRNAs, we noticed that three miRNAs, miR-148a, miR-148b and miR-152, belong to the miRNA-148/152 family, and they can function jointly as a miRNA family [36–38].

Then, to identify the role of the miR-148/152 family members in gastric cancer, we first analyzed the expression of miR-148a, miR-148b, and miR-152 in 52 pairs of clinical gastric cancer tissue samples. As depicted by RT-qPCR, the expression of miR-148a, miR-148b, and miR-152 was lower in gastric cancer tissues than that in adjacent normal tissues (Fig. 4B). In addition, Kaplan–Meier curve analysis results suggested that the overall survival of gastric cancer patients with low expression of miR-148a, miR-148b and miR-152 was shorter than that in patients with high expression of miR-148a, miR-148b and miR-152, respectively (Fig. 4C–E). Pearson analyzed the correlation between miR-148/152 family genes and ITAG5 genes in clinical samples indicated that miR-148/152 family genes were negatively correlated with ITAG5 genes in gastric cancer tissues (Fig. 4F).

Furthermore, RT-qPCR data presented that miR-148a, miR-148b and miR-152 all exhibited lower expression in gastric cancer cells than those in GES-1 cells, with the AGS cells showing the lowest expression of the above three factors (Fig. 4G) and thus selected for subsequent experiments. We also found that the expression of miR-148/152 family genes in CD44^+^EpCAM^+^ cells was lower than that in CD44^−^EpCAM^−^ cells (Fig. 4H). TargetScan database predicted binding sites of the miR-148/152 family members in the 3’UTR of ITGA5 mRNA (Fig. 4I). In addition, the luciferase activity of ITGA5-Wt was reduced in presence of miR-148a mimic, miR-148b mimic or miR-152 mimic while that of ITGA5-Mut was unchanged (Fig. 4J). Western blot results indicated that expression of ITGA5 was diminished in AGS cells treated with miR-148a mimic, miR-148b mimic or miR-152 mimic (Fig. 4K). In brief, low expression of the miR-148/152 family members was detected in the gastric cancer tissues and cells and the miR-148/152 family can target ITGA5 gene.

### ITGA5 overexpression facilitated the cancer stem cell-like traits in CD44^+^EpCAM^+^ cells

**Fig. 5:**
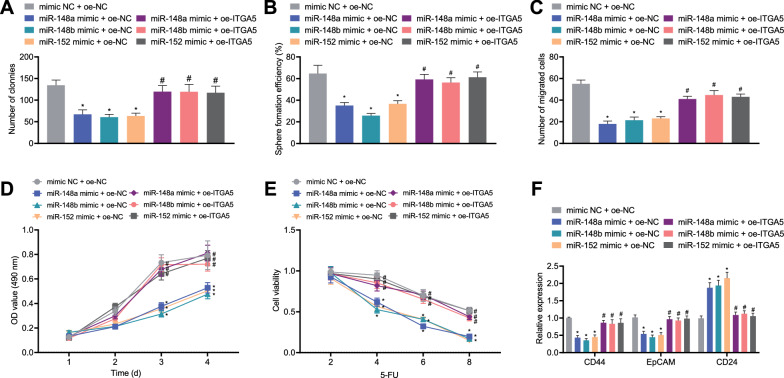
ITGA5 overexpression maintains the gastric cancer stem cell-like traits. **A** Numbers of colonies of CD44^+^EpCAM^+^ cells treated with miR-148a mimic, miR-148b mimic, miR-152 mimic or their combination with ITGA5. **B** Numbers of tumorospheres of CD44^+^EpCAM^+^ cells treated with miR-148a mimic, miR-148b mimic, miR-152 mimic or their combination with ITGA5. **C** Numbers of migrated CD44^+^EpCAM^+^ cells treated with miR-148a mimic, miR-148b mimic, miR-152 mimic or their combination with ITGA5. **D** Viability of CD44^+^EpCAM^+^ cells treated with miR-148a mimic, miR-148b mimic, miR-152 mimic or their combination with ITGA5 measured by CCK-8 assay. **E** Drug resistance of CD44^+^EpCAM^+^ cells treated with miR-148a mimic, miR-148b mimic, miR-152 mimic or their combination with ITGA5 measured by CCK-8 assay. **F** The expression of CD44, EpCAM and CD24 in CD44^+^EpCAM^+^ cells treated with miR-148a mimic, miR-148b mimic, miR-152 mimic or their combination with ITGA5 measured by RT-qPCR. *p < 0.05 compared with mimic NC + oe-NC, ^#^*p* < 0.05 compared with miR-148a mimic + oe-NC/miR-148b mimic + oe-NC/miR-152 mimic + oe-NC. The cell experiment was run in triplicate independently

We found above that ITGA5 was target gene of the miR-148/152 family members. Then, we validated the role of ITGA5 in CD44^+^EpCAM^+^ cells. It was evident that reduced colony formation and sphere-forming abilities were detected upon mimic of miR-148a, miR-148b, and miR-152; while further oe-ITGA5 treatment caused opposing trends (Fig. 5A, B, Additional file 1: Fig. S1A, B). Besides, mimic of miR-148a, miR-148b, and miR-152 led to enhanced cell migration, viability and drug resistance; while further oe-ITGA5 treatment caused opposing trends (Fig. 5C–E, Additional file 1: Fig. S1C). The results of RT-qPCR also showed that mimic of miR-148a, miR-148b, and miR-152 reduced expression of CD44 and EpCAM but elevated CD24 expression; while further oe-ITGA5 treatment caused opposing trends (Fig. 5F). These findings provided evidence of the promotive action of ITGA5 on the stem cell-like traits of CD44^+^EpCAM^+^ cells.

### The miR-148/152 family members suppressed the tumorigenesis of gastric cancer stem cells in vivo

**Fig. 6:**
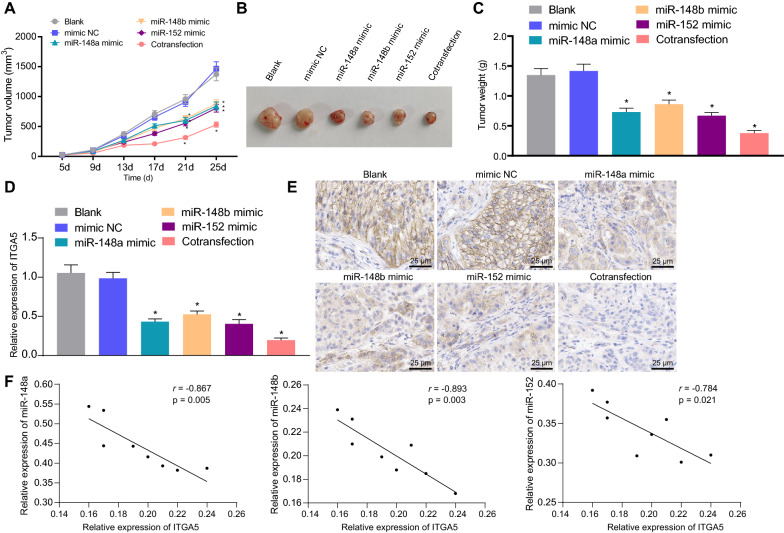
The miR-148/152 family members suppress the tumorigenesis of gastric cancer stem cells in vivo. **A** The volume of xenotransplanted tumors in nude mice treated with miR-148a mimic, miR-148b mimic, and/or miR-152 mimic at the indicated time points. **B** Representative images showing xenografts in nude mice treated with miR-148a mimic, miR-148b mimic, and/or miR-152 mimic. **C** The weight of xenotransplanted tumors in nude mice treated with miR-148a mimic, miR-148b mimic, and/or miR-152 mimic. **D** ITGA5 expression in tissues derived from subcutaneous xenotransplanted tumors in nude mice treated with miR-148a mimic, miR-148b mimic, and/or miR-152 mimic measured by RT-qPCR. **E** Immunohistochemical staining of ITGA5 protein in tissues derived from subcutaneous xenotransplanted tumors in nude mice treated with miR-148a mimic, miR-148b mimic, and/or miR-152 mimic (scale bar: 25 μm). **F** Correlation of miR-148a, miR-148b, and miR-152 expression with ITGA5 expression in tissues derived from subcutaneous xenotransplanted tumors in nude mice analyzed by Pearson’s correlation coefficient. *p < 0.05 compared with mimic-NC

Finally, we established subcutaneous xenotransplanted tumor models of CD44^+^EpCAM (high) cells. As depicted in Fig. 6A–C, overexpression of miR-148a, miR-148b, and miR-152 inhibited the growth of subcutaneous xenotransplanted tumors of gastric cancer stem cells in nude mice. Next, we determined the expression of ITGA5 in tumor tissues derived from subcutaneous xenotransplanted tumors by RT-qPCR and immunohistochemistry (Fig. 6D, E) and found that elevation of the above three factors decreased the expression of ITGA5 in tumor tissues. In addition, the expression of miR-148/152 family members was negatively correlated with that of ITGA5 in tumor tissues (Fig. 6F). Moreover, combined treatment of miR-148a mimic, miR-148b mimic, and miR-152 mimic showed a more profound influence than either treatment alone (Fig. 6A–F). Overall, the miR-148/152 family members exerted inhibitory effects on the tumorigenesis in vivo of gastric cancer stem cells by downregulating ITGA5.

## Discussion

In recent years, gastric cancer stem cells have attracted an increasing amount of research attention due to their potential diagnostic and therapeutic value and implications in immune microenvironment and metastasis [39, 40]. Multiple miRNAs in gastric cancer stem cells appear significant to the development of effective treatment modalities for gastric cancer [41]. In the present investigation, we explored the functional role of the miR-148/152 family members in mediating gastric cancer stem cell-like properties. Our experimental data evinced that the miR-148/152 family members exerted anti-tumor actions by suppressing colony formation, self-renewal and migrative properties, and drug resistance of gastric cancer stem cells through inhibition of ITGA5.

Fundamentally, miR-148a/b and miR-152 were lowly expressed in gastric cancer tissues and cells and were associated with poor prognosis in patients with gastric cancer. The expression of miR-148a has been found to be inhibited by over fourfold in gastric cancer tissues than that in matched non-tumorous tissues, contributing to advanced tumor-node-metastasis stage and lymph node-metastasis, while forced expression of miR-148a corresponds to weakened migrative and invasive properties of malignant cells [42]. Likewise, restored expression of miR-148b has been documented to exert tumor-suppressive effects in gastric cancer by curbing cancer cell proliferation in vitro and tumorigenicity in vivo [43]. An antagonistic function of miR-152 complementation in the mediation of gastric cancer cell proliferation and motility has also been detailed previously [44]. Both miR-148a and miR-152 have been found to be poorly expressed in gastric cancer tissues, which is highly suggestive of their clinical significance in the malignancy [45]. Similar to our finding, low expression of the miR-148/152 family was indicative of poorer oncologic outcomes of patients with hepatocellular carcinoma (HCC) and non-small cell lung cancer, and is considered as a prognostic biomarker [46, 47] that supports our results in gastric cancer.

ITGA5 was observed to be elevated in gastric cancer tissues and cells. Increase of ITGA5 acts importantly in the development of gastric cancer and is considered as a potential therapeutic target and biomarker [10, 48]. The involvement of ITGA5 in miRNA-based modulation has been validated in diverse malignancies, including colorectal cancer, glioblastoma and bladder cancer [49, 50]. For instance, ITGA5 has been implicated in the tumor-suppressive effects of miR-31 on gastric cancer as a target gene to repress tumor cell invasion and metastasis. Targets of deregulated miRNAs have been identified as crucially important to understanding the underlying molecular mechanisms of gastric carcinogenesis in regulating malignant phenotypes [51]. As revealed in our study, ITGA5 was targeted by the miR-148/152 family and attenuated the antitumor potential of miR-148/152 family against gastric cancer. miR-148b has also been found to target ITGA5 to mediate the tumorigenesis [52] of breast cancer. The miR-148/152 family has also been found to enhance breast cancer cell sensitivity to Adriamycin by negatively regulating Spindlin1 [53]. Furthermore, the reported regulatory mechanism appeared to have depressive effects on the biological properties of gastric cancer stem cells including: clone formation, self-renewal and migrative properties, and drug resistance. Given the positive impact of cancer stem cells on self-renewal, differentiation, and tumor formation, targeting cancer stem cells has been proposed as a promising therapeutic modality to improve the prognosis of patients with gastric cancer [54]. Likewise, miR-19b/20a/92a has been shown to facilitate clone formation and self-renewal abilities of CD44^+^EpCAM^+^ cells by targeting E2F transcription factor 1 and homeodomain interacting protein kinase 1 [55]. ITGA5 has been reported to be overexpressed in human mesenchymal stem cells (hMSCs)-treated HCC, and short interfering RNA against ITGA5 blocks the hMSCs-induced migrative and invasive abilities of HCC cells, emphasizing the crucial role of ITGA5 in hMSCs-induced tumor metastasis [56]. Moreover, reduced expression of ITGA5, induced by re-expression of miR-205, reportedly inhibits cancer stem cell-like properties in triple-negative breast cancer [57].

## Conclusions

**Fig. 7:**
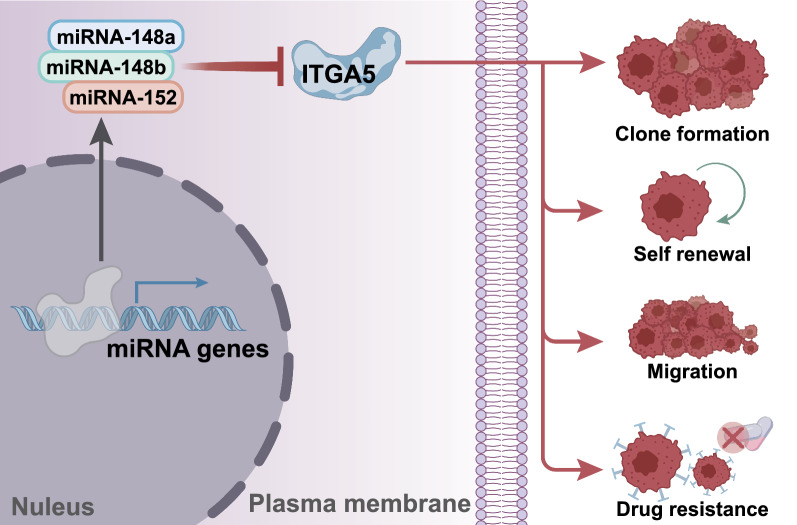
A schematic diagram illustrating the mechanisms underlying the regulation of the miR-148/152 family members in stem cell-like properties in gastric cancer. The miR-148/152 family members target ITGA5 and inhibit its expression, thus reducing self-renewal capacity, clonogenic potential, and drug resistance of gastric cancer stem cells and ultimately suppressing stem cell-like properties in gastric cancer

In summary, the current study detailed the anti-carcinogenic action of the miR-148/152 family members in gastric cancer development through its interplay with ITGA5, thus offering a novel therapeutic target for developing effective treatment modalities against gastric cancer (Fig. 7). Overall, our results also evinced that investigations concerning miRNAs are critical to understanding cancer molecular mechanisms and improving targeted therapies. However, experiments aimed at analytical validation and clinical utility of miRNAs are warranted for the successful translation of miRNAs to be rendered as usable biomarkers.

## Supplementary Information

**Additional file 1: Figure S1.** Representative images of CD44^+^EpCAM^+^ cell clonogenic potential, tumorospheres and migration. **A** Representative images of colonies of CD44^+^EpCAM^+^ cells treated with miR-148a mimic, miR-148b mimic, miR-152 mimic or their combination with ITGA5. **B** Representative images of tumorospheres of CD44^+^EpCAM^+^ cells treated with miR-148a mimic, miR-148b mimic, miR-152 mimic or their combination with ITGA5 (scale bar: 100 μm). **C** Representative images of migration of cells treated with miR-148a mimic, miR-148b mimic, miR-152 mimic or their combination with ITGA5 (scale bar: 50 μm).

**Additional file 2: Table S1.** Clinicopathological characteristics of 52 patients with gastric cancer.

**Additional file 3: Table S2.** Primer sequences for RT-qPCR.

**Additional file 4: Table S3.** Significantly differentially expressed genes.

## Data Availability

The data that support the findings of this study are available from the corresponding author upon reasonable request.

## Abbreviations

miRNA: MicroRNA
ITGA5: Integrin α5
SP: Side population
DEGs: Differentially expressed genes
FBS: Fetal bovine serum
UTR: Untranslated region
ITGA5-Wt: Wild type ITGA5
DMEM: Dulbecco’s modified Eagle’s medium
CCK-8: Cell Counting Kit-8
IgG: Immunoglobulin G
GAPDH: Glyceraldehyde-3-phosphate dehydrogenase
ANOVA: Analysis of variance
