# Supplementary material for: The members of the miR-148/152 family inhibit cancer stem cell-like properties in gastric cancer via negative regulation of ITGA5
Source: J Transl Med. 2023 Feb 10;21:105. doi: 10.1186/s12967-023-03894-1 (PMC9912648; doi:10.1186/s12967-023-03894-1)
Supplement: Supplementary file 2 — Additional file 2: Table S1. Clinicopathological characteristics of 52 patients with gastric cancer. [file 12967_2023_3894_MOESM2_ESM.docx]

**Table S1** Clinicopathological characteristics of 52 patients with gastric cancer

| Characteristics | N |
| --- | --- |
| Age (years) |  |
| ≥ 55 | 17 |
| < 55 | 35 |
| Sex |  |
| Female | 10 |
| Male | 42 |
| TNM staging |  |
| Ⅰ-Ⅱ | 39 |
| Ⅲ-Ⅳ | 13 |
| Tumor size (cm) |  |
| < 5 | 36 |
| ≥ 5 | 16 |
| Differentiation |  |
| Well-moderate | 40 |
| Poor | 12 |
| Lymphatic metastasis |  |
| No | 24 |
| Yes | 28 |
| Remote metastasis |  |
| No | 47 |
| Yes | 5 |
