# Supplementary material for: The members of the miR-148/152 family inhibit cancer stem cell-like properties in gastric cancer via negative regulation of ITGA5
Source: J Transl Med. 2023 Feb 10;21:105. doi: 10.1186/s12967-023-03894-1 (PMC9912648; doi:10.1186/s12967-023-03894-1)
Supplement: Supplementary file 3 — Additional file 3: Table S2. Primer sequences for RT-qPCR. [file 12967_2023_3894_MOESM3_ESM.docx]

**Table S2** Primer sequences for RT-qPCR.

| Gene | Sequences |
| --- | --- |
| CD44 | Forward: 5′-CGGACACCATGGACAAGTTT-3’  Reverse: 5′-AGCTTTTTCTTCTGCCCACA-3’ |
| CD133 | Forward: 5′-TAGGAGGCGGAATTCTTGAC-3′  Reverse: 5′-AGAGATGACCGCAGGCTAGT-3′ |
| MDR1 | Forward: 5′-GAGGAAGACATGACCAGGTA-3’  Reverse: 5′-CTGTCGCATTATAGCATGAA-3’ |
| EpCAM | Forward: 5′-CTGCCAAATGTTTGGTGATG-3’  Reverse: 5′-ACGCGTTGTGATCTCCTTCT-3’ |
| Oct4 | Forward: 5′-AAGGAGAAGCTGGAGCAA-3′  Reverse: 5′-GAGGGTTTCTGCTTTGCAT-3′ |
| miR-148a | Forward: 5′-TCAGTGCACTACAGAACTTTGT-3′  Reverse: Reverse universal primer |
| miR-152 | Forward: 5′-TCAGTGCATGACAGAACTTGG-3′  Reverse: Reverse universal primer |
| miR-148b | Forward: 5′-AAGTTCTGTTATACACTCAGGC-3′  Reverse: Reverse universal primer |
| U6 | Forward: 5′-CTCGCTTCGGCAGCACA-3′  Reverse: Reverse universal primer |
| ABCG2 | Forward: 5′-GGGTTCTCTTCTTCCTGACGACC-3’  Reverse: 5′-TGGTTGTGAGATTGACCAACAGACC-3’ |
| GAPDH | Forward: 5′-AACGGATTTGGTCGTATTGGG-3′  Reverse: 5′-TCGCTCCTGGAAGATGGTGAT-3′ |
| CD24 | Forward: 5′-GCGCTCACAGAACAAAGCAA-3′  Reverse: 5′-AGACGCCATTTGGATTGGGT-3 |
| ITGA5 | Forward: 5′-TAATACCAGCCAGCCAGGAGTG-3  Reverse: 5′-TGTCAAATTCAATGGGGGTGC-3 |

Note: RT-qPCR, reverse transcription quantitative polymerase chain reaction; MDR1, multiple drug resistance 1; miR, microRNA; ABCG2, ATP-binding cassette transporter subfamily G member 2; GAPDH, glyceraldehyde-3-phosphate dehydrogenase; ITGA5, integrin alpha-5.
