# Supplementary material for: The members of the miR-148/152 family inhibit cancer stem cell-like properties in gastric cancer via negative regulation of ITGA5
Source: J Transl Med. 2023 Feb 10;21:105. doi: 10.1186/s12967-023-03894-1 (PMC9912648; doi:10.1186/s12967-023-03894-1)
Supplement: Supplementary file 4 — Additional file 4: Table S3. Significantly differentially expressed genes. [file 12967_2023_3894_MOESM4_ESM.docx]

**Table S3** Significantly differentially expressed genes

| Tag | logFC | adj.P.Val |
| --- | --- | --- |
| RNASE1 | -11.2631 | 4.80E-23 |
| RAB27A | -9.57442 | 4.80E-23 |
| ATP4B | -11.8765 | 2.10E-22 |
| GIF | -13.4964 | 1.11E-21 |
| DGKD | -8.79419 | 7.63E-20 |
| SSR4 | -10.982 | 8.41E-20 |
| ALDH2 | -10.6234 | 8.41E-20 |
| ADH7 | -7.88952 | 2.63E-19 |
| CAPN2 | -9.95891 | 3.65E-19 |
| CA2 | -12.1447 | 7.10E-19 |
| ATP1B3 | 9.131805 | 7.10E-19 |
| CTSE | -11.9451 | 8.31E-19 |
| MEST | 9.20781 | 2.73E-18 |
| ATP4A | -12.3919 | 2.76E-17 |
| IGJ | -12.3548 | 5.36E-17 |
| ALDH6A1 | -8.8594 | 5.53E-17 |
| ALDH3A1 | -10.2731 | 6.08E-17 |
| RAB27B | -8.72458 | 1.54E-16 |
| CBX3 | 8.6962 | 2.62E-16 |
| HERPUD1 | -9.71194 | 4.86E-16 |
| KLF4 | -10.0183 | 5.94E-16 |
| HADH | -8.98779 | 7.02E-16 |
| NFE2L2 | -9.17849 | 7.73E-16 |
| GLUL | -10.2983 | 1.28E-15 |
| HPGD | -10.7765 | 2.07E-15 |
| SCNN1G | -7.12246 | 2.51E-15 |
| SCNN1B | -7.85547 | 2.55E-15 |
| ETFDH | -7.561 | 3.47E-15 |
| FN1 | 10.49367 | 3.54E-15 |
| GATA6 | -9.11087 | 3.60E-15 |
| FBP2 | -7.41045 | 4.87E-15 |
| MUC5AC | -11.8222 | 6.48E-15 |
| LIFR | -8.37201 | 1.15E-14 |
| CHGA | -10.6855 | 1.21E-14 |
| ADRB2 | -7.12584 | 1.21E-14 |
| PGC | -13.7414 | 1.33E-14 |
| FCGBP | -11.0892 | 1.35E-14 |
| CPA2 | -10.623 | 1.61E-14 |
| PPAP2B | -9.03112 | 1.61E-14 |
| FMO4 | -7.32067 | 1.92E-14 |
| CA9 | -10.1027 | 1.96E-14 |
| CYP2C9 | -7.90709 | 2.00E-14 |
| KCNJ15 | -7.74521 | 2.00E-14 |
| S100A10 | 10.87321 | 2.00E-14 |
| AMPD1 | -6.96264 | 2.75E-14 |
| NQO1 | -10.5471 | 2.85E-14 |
| LIPF | -13.9798 | 2.99E-14 |
| AKR1C1 | -9.62223 | 3.39E-14 |
| IQGAP2 | -8.87341 | 3.39E-14 |
| RNASE4 | -9.83086 | 3.71E-14 |
| REG1A | -12.2529 | 3.90E-14 |
| SULT1C2 | -10.1485 | 3.90E-14 |
| ALDH1A1 | -11.0787 | 3.91E-14 |
| COL6A3 | 10.91188 | 4.57E-14 |
| XBP1 | -9.79775 | 5.87E-14 |
| AQP4 | -8.70662 | 5.87E-14 |
| SEPP1 | -10.3574 | 9.04E-14 |
| CYB5A | -9.47266 | 9.04E-14 |
| LTF | -11.6532 | 1.04E-13 |
| ATP5O | -9.0272 | 1.21E-13 |
| CKM | -7.83805 | 1.22E-13 |
| UGCG | -9.31278 | 1.23E-13 |
| ELL2 | -8.40946 | 1.47E-13 |
| MAL | -9.33766 | 1.74E-13 |
| HDC | -7.62418 | 1.82E-13 |
| GPX3 | -9.91646 | 2.58E-13 |
| CKMT2 | -8.48548 | 2.67E-13 |
| LBR | 9.040469 | 3.28E-13 |
| TFF2 | -13.0247 | 3.46E-13 |
| TFF1 | -13.4139 | 3.86E-13 |
| AKR1C3 | -11.2834 | 4.14E-13 |
| GCNT2 | -7.18767 | 4.22E-13 |
| HMGCL | -7.66411 | 4.58E-13 |
| SH3GL2 | -7.55014 | 4.72E-13 |
| SLC9A1 | -7.81409 | 6.44E-13 |
| CCKBR | -8.55889 | 8.42E-13 |
| TUBB | 9.834238 | 8.42E-13 |
| ARPC1B | 9.75742 | 9.64E-13 |
| HSD17B4 | -7.96085 | 1.32E-12 |
| TEAD4 | 7.172456 | 1.42E-12 |
| MDH1 | -10.1156 | 1.90E-12 |
| SELENBP1 | -9.40131 | 2.39E-12 |
| COL1A2 | 11.06169 | 2.55E-12 |
| UBE2S | 8.454378 | 2.82E-12 |
| MT1E | -11.152 | 3.42E-12 |
| TGIF1 | 7.177794 | 3.42E-12 |
| PTPRN2 | -7.80533 | 4.08E-12 |
| BMP6 | -4.9593 | 4.08E-12 |
| CTSB | 9.90115 | 4.08E-12 |
| MT1G | -10.9545 | 4.86E-12 |
| HSPD1 | 9.276503 | 5.01E-12 |
| KRT20 | -11.3257 | 5.02E-12 |
| RCN1 | 9.041832 | 5.07E-12 |
| UGT2B15 | -8.91131 | 5.87E-12 |
| CYP2C18 | -9.33608 | 6.11E-12 |
| RPL8 | 11.00076 | 6.67E-12 |
| IRX5 | -5.69265 | 6.97E-12 |
| IGFBP2 | -10.6236 | 1.01E-11 |
| EPOR | -6.59336 | 1.01E-11 |
| PDIA2 | -7.31073 | 1.06E-11 |
| CKB | -10.1996 | 1.14E-11 |
| TIMP1 | 11.1486 | 1.35E-11 |
| S100P | -12.218 | 1.59E-11 |
| IGFALS | -6.71572 | 1.73E-11 |
| GALE | -7.51543 | 1.94E-11 |
| ID4 | -7.43489 | 1.97E-11 |
| ABLIM1 | -8.7943 | 2.02E-11 |
| SCP2 | -9.3748 | 2.05E-11 |
| HOXC6 | 7.970769 | 2.27E-11 |
| BMP5 | -5.18517 | 2.65E-11 |
| SLC25A46 | -7.85167 | 2.72E-11 |
| SEL1L | -7.42649 | 2.82E-11 |
| TRIP11 | -5.96006 | 2.99E-11 |
| SLC1A2 | -6.2149 | 3.28E-11 |
| INHBA | 8.127357 | 3.61E-11 |
| IRF4 | -5.82164 | 3.86E-11 |
| SNRPG | 9.699608 | 4.09E-11 |
| MIF | 10.61282 | 4.09E-11 |
| NR0B2 | -6.89338 | 5.46E-11 |
| HADHB | -9.49971 | 5.68E-11 |
| NME1 | 9.455863 | 5.68E-11 |
| MT2A | -11.3861 | 6.57E-11 |
| SLC9A2 | -5.91373 | 6.57E-11 |
| TNFRSF17 | -8.97425 | 6.63E-11 |
| CD81 | 9.916757 | 6.63E-11 |
| FAP | 7.84551 | 6.86E-11 |
| PHKB | -6.69164 | 6.88E-11 |
| MUT | -7.72919 | 7.25E-11 |
| PRKDC | 7.671794 | 7.52E-11 |
| HSPB1 | 10.00285 | 9.15E-11 |
| ADAR | 8.924891 | 9.28E-11 |
| KPNA2 | 9.365297 | 9.97E-11 |
| COL3A1 | 10.90103 | 1.02E-10 |
| COL4A1 | 9.809143 | 1.03E-10 |
| HOXB7 | 8.169017 | 1.15E-10 |
| ACADM | -8.97823 | 1.18E-10 |
| LPGAT1 | 7.448652 | 1.18E-10 |
| GINS1 | 8.090611 | 1.30E-10 |
| ATP5A1 | -9.56952 | 1.31E-10 |
| NNT | -7.4115 | 1.31E-10 |
| CTNNB1 | 8.237531 | 1.31E-10 |
| S100A11 | 10.96627 | 1.31E-10 |
| SNRPD2 | 9.909245 | 1.37E-10 |
| RORC | -6.68832 | 1.46E-10 |
| DRD5 | -7.56497 | 1.47E-10 |
| GPT | -6.01991 | 1.62E-10 |
| GPR64 | -7.63856 | 1.80E-10 |
| LPIN2 | -7.90761 | 1.81E-10 |
| BTD | -5.87577 | 2.06E-10 |
| ATP5F1 | -9.26364 | 2.09E-10 |
| FMO5 | -7.26005 | 2.16E-10 |
| TLE4 | -5.99779 | 2.50E-10 |
| SST | -10.5552 | 3.22E-10 |
| UBE2I | 6.708336 | 3.22E-10 |
| SNRPB | 9.591043 | 3.35E-10 |
| KIT | -7.965 | 3.44E-10 |
| PPP2R5A | -7.15493 | 3.44E-10 |
| ACO1 | -7.20531 | 3.45E-10 |
| COL1A1 | 10.75048 | 3.52E-10 |
| SCNN1A | -8.45599 | 3.75E-10 |
| COL18A1 | 7.528046 | 3.89E-10 |
| FUCA1 | -8.56913 | 4.13E-10 |
| ESRRB | -4.77369 | 4.39E-10 |
| ABCD3 | -8.29546 | 4.53E-10 |
| NPY | -6.11358 | 4.53E-10 |
| LGALS1 | 10.46007 | 4.53E-10 |
| MFAP2 | 8.204239 | 4.71E-10 |
| COX4I1 | -9.1354 | 4.93E-10 |
| BLOC1S1 | -8.10395 | 5.05E-10 |
| CIRBP | -7.69625 | 5.05E-10 |
| BMP1 | 5.024675 | 5.05E-10 |
| PDE4A | -5.42135 | 5.27E-10 |
| SSTR1 | -8.29152 | 5.57E-10 |
| REG3A | -10.8127 | 6.34E-10 |
| ITPKA | -6.4104 | 6.61E-10 |
| APLP1 | -8.05419 | 6.99E-10 |
| SNRPE | 8.161784 | 7.56E-10 |
| ADA | -8.30859 | 9.14E-10 |
| GCKR | -6.18409 | 9.83E-10 |
| FBP1 | -8.71347 | 9.95E-10 |
| SERPINH1 | 9.073302 | 1.19E-09 |
| TST | -9.82817 | 1.20E-09 |
| IL32 | 9.287702 | 1.23E-09 |
| PCNA | 9.507083 | 1.23E-09 |
| COL4A2 | 9.339127 | 1.48E-09 |
| CD59 | -8.93167 | 1.60E-09 |
| PAFAH2 | -5.80477 | 1.60E-09 |
| PUF60 | 8.478222 | 1.60E-09 |
| PSMB3 | 9.549438 | 1.61E-09 |
| SPARC | 10.35284 | 1.61E-09 |
| NCOA4 | -9.28169 | 1.73E-09 |
| UPP1 | 6.891795 | 1.75E-09 |
| PSMB1 | 8.747966 | 1.77E-09 |
| GPX1 | 9.480335 | 1.81E-09 |
| TMEM131 | -7.62769 | 1.89E-09 |
| PSMD4 | 8.216037 | 1.89E-09 |
| MTMR3 | -5.31606 | 1.92E-09 |
| ATP2A3 | -7.49304 | 2.00E-09 |
| UFD1L | 8.097204 | 2.23E-09 |
| SPINK1 | -11.6804 | 2.45E-09 |
| MT1X | -10.9675 | 2.58E-09 |
| AUH | -8.54792 | 2.59E-09 |
| AZGP1 | -9.95393 | 2.67E-09 |
| IFITM1 | 11.46198 | 2.80E-09 |
| ANG | -9.13912 | 2.80E-09 |
| CCT6A | 8.610982 | 3.20E-09 |
| MT1F | -10.6986 | 3.49E-09 |
| ACTN1 | 9.007416 | 3.57E-09 |
| HSPE1 | 9.694189 | 3.57E-09 |
| SSB | 8.31979 | 3.63E-09 |
| C5 | -6.99374 | 3.66E-09 |
| FOLR1 | -8.69045 | 3.74E-09 |
| LRP8 | 6.274337 | 3.75E-09 |
| HSPG2 | 7.863099 | 3.97E-09 |
| SPINK2 | -6.29368 | 4.03E-09 |
| IDUA | -4.99535 | 4.03E-09 |
| SLC25A4 | -7.52139 | 4.10E-09 |
| MAGI1 | -5.60591 | 4.12E-09 |
| ACADL | -5.38266 | 4.27E-09 |
| FBL | 9.370577 | 4.27E-09 |
| XK | -8.36703 | 4.38E-09 |
| PTPRZ1 | -7.97014 | 4.61E-09 |
| HSP90AA1 | 10.14571 | 4.64E-09 |
| THY1 | 8.239452 | 4.84E-09 |
| RCC1 | 7.024055 | 4.98E-09 |
| CD9 | 9.188996 | 4.99E-09 |
| NID2 | 7.878269 | 5.52E-09 |
| ASPA | -5.45924 | 5.67E-09 |
| AADAC | -9.14211 | 5.68E-09 |
| MT1H | -11.204 | 5.92E-09 |
| CHAD | -4.93596 | 5.95E-09 |
| ALDH3A2 | -8.25252 | 6.03E-09 |
| TBCB | 8.363712 | 6.08E-09 |
| UBE2C | 9.323976 | 6.56E-09 |
| PSMA1 | 8.932736 | 6.78E-09 |
| RAB5B | -7.57685 | 7.34E-09 |
| PFN1 | 10.09236 | 7.34E-09 |
| AKR1A1 | -8.6202 | 7.56E-09 |
| CSTA | -8.93001 | 7.66E-09 |
| AMT | -7.05243 | 7.70E-09 |
| OXCT1 | -8.87624 | 7.86E-09 |
| CYP2C19 | -5.72724 | 7.88E-09 |
| GADD45A | -8.7449 | 8.27E-09 |
| NCL | 8.615291 | 9.24E-09 |
| FUT1 | -7.13687 | 9.36E-09 |
| HSP90AB1 | 10.17161 | 9.48E-09 |
| PBXIP1 | -7.45373 | 9.64E-09 |
| PTPN12 | 7.372791 | 1.01E-08 |
| ACADVL | -8.59544 | 1.03E-08 |
| KIF23 | 6.103533 | 1.12E-08 |
| RNPS1 | 8.055472 | 1.14E-08 |
| ACOT7 | 7.307312 | 1.18E-08 |
| LOXL1 | 7.266582 | 1.19E-08 |
| RAD21 | 8.654073 | 1.25E-08 |
| FGA | -7.78465 | 1.36E-08 |
| MITF | -7.16263 | 1.43E-08 |
| BHMT | -6.04755 | 1.44E-08 |
| ALCAM | -7.90131 | 1.46E-08 |
| CKS2 | 9.41488 | 1.48E-08 |
| LGALS9 | -8.13376 | 1.52E-08 |
| CBX1 | 8.188675 | 1.54E-08 |
| CCNG1 | -9.12245 | 1.58E-08 |
| CBR1 | -8.39303 | 1.61E-08 |
| PLA2G1B | -6.04059 | 1.69E-08 |
| CHKB | -6.86981 | 1.81E-08 |
| RPS19 | 10.47297 | 1.96E-08 |
| UBB | -11.0862 | 2.00E-08 |
| ESM1 | 5.994824 | 2.03E-08 |
| HERC1 | -5.67531 | 2.09E-08 |
| RAP1GAP | -8.18347 | 2.13E-08 |
| PTTG1IP | -9.42538 | 2.18E-08 |
| UBE4A | -8.35048 | 2.18E-08 |
| CD36 | -6.8805 | 2.37E-08 |
| HK1 | -7.59816 | 2.47E-08 |
| ENO1 | 9.113872 | 2.49E-08 |
| ATP13A3 | 7.042442 | 2.59E-08 |
| NR3C2 | -7.51257 | 2.65E-08 |
| RAB11A | -8.24728 | 2.80E-08 |
| SLCO2A1 | -7.96757 | 2.92E-08 |
| RGS7 | -5.1251 | 2.92E-08 |
| ACAT1 | -8.0651 | 3.39E-08 |
| NASP | 7.028429 | 3.42E-08 |
| NTHL1 | 6.329555 | 3.47E-08 |
| MYL4 | -4.88205 | 3.72E-08 |
| ID3 | -8.80294 | 3.77E-08 |
| CDC25B | 8.436051 | 4.01E-08 |
| SOX2 | -5.90881 | 4.41E-08 |
| RRS1 | 7.557232 | 4.50E-08 |
| COL4A5 | -6.93164 | 4.51E-08 |
| ITGB1 | 8.05873 | 4.53E-08 |
| LOXL2 | 6.385918 | 4.71E-08 |
| STIM1 | -5.57683 | 4.79E-08 |
| EYA2 | -7.06595 | 4.93E-08 |
| DNASE1 | -4.41197 | 4.96E-08 |
| FMOD | -9.37052 | 5.22E-08 |
| ID1 | -9.88899 | 5.33E-08 |
| UGP2 | -7.84756 | 5.59E-08 |
| CHN1 | 6.707609 | 5.88E-08 |
| REEP5 | -8.4284 | 6.22E-08 |
| TUBA1A | 9.884853 | 6.43E-08 |
| AP2S1 | 8.361373 | 6.52E-08 |
| TCEB1 | 8.425385 | 6.92E-08 |
| LDHA | 10.49141 | 6.97E-08 |
| KIAA0141 | -5.84186 | 7.24E-08 |
| XPA | -6.93213 | 7.32E-08 |
| DAP3 | 7.04013 | 7.51E-08 |
| GPD1L | -8.39936 | 7.58E-08 |
| COX7B | -9.34727 | 7.81E-08 |
| TOP1 | 7.926781 | 7.81E-08 |
| SURF1 | -7.44693 | 7.97E-08 |
| ROR1 | -6.3041 | 8.07E-08 |
| HPCAL1 | -7.57024 | 8.10E-08 |
| KIF2C | 6.855129 | 8.22E-08 |
| TMSB10 | 10.95301 | 8.22E-08 |
| CAD | 6.377575 | 8.60E-08 |
| HYAL1 | -7.75919 | 9.15E-08 |
| COL2A1 | -7.1458 | 9.39E-08 |
| SMS | 8.364192 | 9.64E-08 |
| CCT7 | 8.460376 | 9.66E-08 |
| SMYD5 | 5.870163 | 9.67E-08 |
| ZNF85 | -6.60781 | 1.08E-07 |
| PMM1 | -6.22951 | 1.08E-07 |
| PRDX1 | 9.771831 | 1.08E-07 |
| WASF1 | 6.176212 | 1.10E-07 |
| PTGER3 | -6.44991 | 1.11E-07 |
| HMGA1 | 7.891047 | 1.12E-07 |
| VARS | 6.583918 | 1.17E-07 |
| PRKACB | -7.98064 | 1.26E-07 |
| RBL2 | -7.43484 | 1.29E-07 |
| HTR1E | -5.66847 | 1.31E-07 |
| KLF9 | -6.66811 | 1.32E-07 |
| NOLC1 | 7.303418 | 1.33E-07 |
| MYOC | -7.804 | 1.44E-07 |
| COL5A2 | 9.525945 | 1.44E-07 |
| KIF14 | 6.770074 | 1.46E-07 |
| ITGB1BP1 | 6.175302 | 1.49E-07 |
| ZNF211 | -5.92874 | 1.50E-07 |
| KL | -6.15641 | 1.51E-07 |
| GOT1 | -8.28941 | 1.55E-07 |
| CCT5 | 8.319705 | 1.57E-07 |
| GFRA2 | -3.74748 | 1.58E-07 |
| NEK2 | 7.159609 | 1.60E-07 |
| CKS1B | 9.020624 | 1.69E-07 |
| FAM110B | -5.31618 | 1.85E-07 |
| CYFIP2 | -7.74195 | 1.89E-07 |
| MLH1 | -7.74774 | 2.04E-07 |
| MUC1 | -10.1396 | 2.07E-07 |
| THBS2 | 9.70583 | 2.07E-07 |
| TEF | -4.99313 | 2.11E-07 |
| BYSL | 7.11917 | 2.25E-07 |
| ALAD | -7.1386 | 2.28E-07 |
| MIA3 | -6.4286 | 2.30E-07 |
| PSMD11 | 7.581778 | 2.30E-07 |
| ACO2 | -8.2673 | 2.30E-07 |
| DBT | -6.8443 | 2.62E-07 |
| CDK4 | 8.652406 | 2.63E-07 |
| DCUN1D4 | -5.57434 | 2.66E-07 |
| SMPDL3B | -6.17943 | 2.68E-07 |
| COL4A6 | -5.12874 | 2.69E-07 |
| ASAH1 | 7.944504 | 2.69E-07 |
| PNOC | -6.44225 | 2.71E-07 |
| TBL3 | 5.320927 | 2.71E-07 |
| BCL2L2 | -6.18806 | 2.78E-07 |
| ISL1 | -7.10765 | 2.80E-07 |
| CLUL1 | -5.26625 | 2.82E-07 |
| TUBB3 | 9.138265 | 2.94E-07 |
| GPX4 | 9.016227 | 3.11E-07 |
| ARSD | -7.02031 | 3.20E-07 |
| ZER1 | -4.59176 | 3.22E-07 |
| MAP3K5 | -7.4646 | 3.42E-07 |
| LY6E | 9.373427 | 3.42E-07 |
| PEX7 | -7.20681 | 3.43E-07 |
| IGBP1 | -7.72759 | 3.47E-07 |
| ZYX | 7.928437 | 3.50E-07 |
| CDC6 | 6.443267 | 3.51E-07 |
| MYBL2 | 6.973165 | 3.58E-07 |
| PPP1CB | -8.64488 | 3.61E-07 |
| KRT18 | 10.73008 | 3.77E-07 |
| TGFBI | 9.544834 | 3.90E-07 |
| NAE1 | 8.17596 | 3.92E-07 |
| RAE1 | 7.344321 | 4.02E-07 |
| LIF | 7.162787 | 4.15E-07 |
| PTPRH | -7.44658 | 4.28E-07 |
| OPCML | -4.75913 | 4.31E-07 |
| ASS1 | 10.23288 | 4.33E-07 |
| SNX19 | -5.21033 | 4.51E-07 |
| CD79A | -8.43127 | 4.54E-07 |
| NDUFA5 | -7.97906 | 4.60E-07 |
| ETV4 | 6.540746 | 4.60E-07 |
| GPI | 9.328728 | 4.68E-07 |
| LDHB | -10.4057 | 4.68E-07 |
| NUCB2 | -8.18517 | 4.70E-07 |
| CPNE1 | 7.983454 | 4.76E-07 |
| STIL | 7.539572 | 4.85E-07 |
| TCEB3 | -6.60416 | 5.02E-07 |
| S100A3 | 5.344875 | 5.06E-07 |
| QSOX1 | -7.28891 | 5.10E-07 |
| SPP1 | 9.85499 | 5.16E-07 |
| IFI6 | 9.1557 | 5.21E-07 |
| UMPS | 6.118258 | 5.38E-07 |
| CHI3L1 | 7.619132 | 5.39E-07 |
| SULT2A1 | -7.73204 | 5.40E-07 |
| EZH2 | 7.086976 | 5.41E-07 |
| C3 | 10.44282 | 5.64E-07 |
| ME1 | -8.13398 | 5.70E-07 |
| PRKCD | -6.83059 | 5.97E-07 |
| ISG15 | 9.703284 | 6.05E-07 |
| RAB31 | 8.912814 | 6.21E-07 |
| DGKZ | 5.964057 | 6.38E-07 |
| FLAD1 | 6.193721 | 6.47E-07 |
| NUP62 | 6.423479 | 6.47E-07 |
| TXNIP | -9.80288 | 6.48E-07 |
| COX6A1 | -10.0003 | 6.55E-07 |
| XRCC6 | 8.186259 | 6.61E-07 |
| MCM2 | 8.220908 | 6.62E-07 |
| GCLC | -6.89439 | 6.78E-07 |
| TSC22D3 | -7.90504 | 7.12E-07 |
| SMAD7 | -6.88204 | 7.12E-07 |
| ITGA5 | 7.935331 | 7.25E-07 |
| DHCR24 | -8.77465 | 7.50E-07 |
| HMOX2 | -6.7215 | 8.07E-07 |
| PRMT1 | 7.95706 | 8.22E-07 |
| CRADD | -6.13281 | 8.41E-07 |
| YWHAQ | 8.961087 | 8.48E-07 |
| AFF3 | -4.31527 | 8.54E-07 |
| PSMB4 | 8.661755 | 8.54E-07 |
| ATP5J | -9.62305 | 8.59E-07 |
| PAK1 | -5.85167 | 8.59E-07 |
| DNAH1 | -3.02056 | 8.82E-07 |
| CDC123 | 8.262083 | 9.03E-07 |
| GHR | -7.04729 | 9.24E-07 |
| HPN | -6.95775 | 9.46E-07 |
| NUP98 | 6.100004 | 9.46E-07 |
| ATIC | 8.404377 | 9.62E-07 |
| DHX9 | 5.883975 | 9.71E-07 |
| SNRPF | 8.985256 | 9.79E-07 |
| SLC25A5 | -10.013 | 9.82E-07 |
| EIF4A3 | 8.481582 | 1.00E-06 |
| LRRC17 | -6.78656 | 1.05E-06 |
| PSMC1 | 7.973311 | 1.08E-06 |
| UQCRC2 | -8.45866 | 1.14E-06 |
| PFDN4 | 7.666189 | 1.15E-06 |
| SDCBP | -9.54264 | 1.18E-06 |
| DMD | -5.76323 | 1.19E-06 |
| EFNA3 | 6.665722 | 1.20E-06 |
| FNTA | 7.668147 | 1.23E-06 |
| PSMD2 | 8.264016 | 1.24E-06 |
| SNRPD1 | 7.976131 | 1.27E-06 |
| MMP12 | 10.16809 | 1.28E-06 |
| PPP2R3A | -7.66799 | 1.28E-06 |
| APOBEC1 | -7.25097 | 1.28E-06 |
| BCAP31 | 8.503532 | 1.30E-06 |
| P4HB | -9.51244 | 1.33E-06 |
| DNM1 | 5.872156 | 1.34E-06 |
| CBS | -6.01478 | 1.37E-06 |
| CSE1L | 8.609866 | 1.37E-06 |
| MVK | -4.88738 | 1.38E-06 |
| OSMR | 7.252072 | 1.38E-06 |
| EFNB3 | -5.20212 | 1.39E-06 |
| SAFB | 5.391687 | 1.40E-06 |
| VCL | 7.617678 | 1.41E-06 |
| MARCKS | -8.24658 | 1.41E-06 |
| ME2 | -7.45842 | 1.42E-06 |
| MS4A2 | -5.2182 | 1.42E-06 |
| RRM2 | 8.96748 | 1.47E-06 |
| ABCC8 | -3.6472 | 1.48E-06 |
| ARAF | -6.33495 | 1.53E-06 |
| GNAT1 | -4.01886 | 1.54E-06 |
| COL9A2 | -6.82531 | 1.54E-06 |
| WDR43 | 6.916796 | 1.54E-06 |
| ILF3 | 6.531674 | 1.56E-06 |
| RFC3 | 6.528652 | 1.66E-06 |
| MCM4 | 6.764817 | 1.71E-06 |
| COL11A1 | 8.356635 | 1.71E-06 |
| PURA | -6.40145 | 1.72E-06 |
| BLVRB | -8.71561 | 1.72E-06 |
| P2RY14 | -7.26486 | 1.76E-06 |
| NR3C1 | -6.98696 | 1.76E-06 |
| CST4 | 7.995658 | 1.76E-06 |
| RPS6KA2 | -6.07963 | 1.79E-06 |
| CCNO | -6.06636 | 1.81E-06 |
| LMAN1 | -7.45937 | 1.82E-06 |
| ADAM17 | 6.00706 | 1.82E-06 |
| SNRPD3 | 8.416371 | 1.83E-06 |
| HTATSF1 | 6.65168 | 1.92E-06 |
| PAFAH1B3 | 7.104659 | 1.92E-06 |
| AMPD2 | 5.906255 | 1.93E-06 |
| BNIP3 | -7.88385 | 1.96E-06 |
| AFAP1 | 5.600433 | 1.98E-06 |
| GUCY1B3 | 6.73352 | 1.98E-06 |
| CSTF1 | 6.3223 | 1.99E-06 |
| CDC20 | 7.834932 | 1.99E-06 |
| CKAP5 | 7.126254 | 2.06E-06 |
| EIF3E | 8.753594 | 2.08E-06 |
| GCM1 | -4.07239 | 2.13E-06 |
| HGD | -5.8082 | 2.13E-06 |
| CNN2 | 7.130909 | 2.19E-06 |
| KIAA0125 | -5.8369 | 2.21E-06 |
| CD97 | 7.623019 | 2.23E-06 |
| GLA | 7.497269 | 2.25E-06 |
| NCBP2 | 6.841882 | 2.32E-06 |
| ACSM3 | -7.07835 | 2.35E-06 |
| STMN1 | 6.237462 | 2.35E-06 |
| HDLBP | -6.92463 | 2.37E-06 |
| GRIA3 | -3.17349 | 2.37E-06 |
| MAZ | 6.558198 | 2.37E-06 |
| PSMC3IP | 5.133358 | 2.42E-06 |
| FRY | -5.64533 | 2.46E-06 |
| MKI67 | 7.034496 | 2.46E-06 |
| NCAM1 | -4.87321 | 2.49E-06 |
| MAN2A1 | -7.30097 | 2.53E-06 |
| FBN2 | -4.10593 | 2.54E-06 |
| PGK1 | 8.423935 | 2.59E-06 |
| MAPRE1 | 8.229358 | 2.60E-06 |
| SERPINI1 | -6.89936 | 2.74E-06 |
| KIAA0513 | -5.07881 | 2.78E-06 |
| SHFM1 | 8.569989 | 2.81E-06 |
| AMHR2 | -3.75417 | 2.82E-06 |
| BTG2 | -7.83655 | 2.83E-06 |
| ISCU | -9.3216 | 2.86E-06 |
| MMP11 | 7.091915 | 2.86E-06 |
| HIF1A | 8.886998 | 2.95E-06 |
| MAD2L1 | 7.798814 | 2.98E-06 |
| MAP4 | 6.21108 | 3.00E-06 |
| P2RY4 | -5.11777 | 3.05E-06 |
| TOP2A | 8.365866 | 3.06E-06 |
| PDLIM7 | 5.022242 | 3.08E-06 |
| ANGPT1 | -4.99522 | 3.12E-06 |
| SYN2 | -4.56387 | 3.12E-06 |
| TPD52L1 | -8.1208 | 3.16E-06 |
| STK3 | 6.960332 | 3.24E-06 |
| CCL4 | 7.577976 | 3.31E-06 |
| LY9 | -6.24397 | 3.32E-06 |
| HTRA1 | 8.778098 | 3.40E-06 |
| LMO4 | -6.83784 | 3.41E-06 |
| PDHA1 | -8.25404 | 3.53E-06 |
| NMT1 | 6.069576 | 3.55E-06 |
| SF3B3 | 7.185784 | 3.71E-06 |
| EDNRA | 6.81445 | 3.71E-06 |
| RANBP1 | 7.836214 | 3.73E-06 |
| SLC39A6 | 6.850323 | 3.82E-06 |
| RHEB | 6.910481 | 3.82E-06 |
| PTPRG | 5.414941 | 3.92E-06 |
| RIMS3 | -5.29789 | 3.98E-06 |
| LYST | -5.15074 | 3.98E-06 |
| PSMA4 | 8.928909 | 3.98E-06 |
| KARS | 8.943645 | 4.10E-06 |
| TCN1 | -10.3966 | 4.13E-06 |
| TRIM29 | 8.167636 | 4.18E-06 |
| TGFB1 | 6.055806 | 4.19E-06 |
| EIF3B | 7.43102 | 4.21E-06 |
| COX7C | -9.23612 | 4.22E-06 |
| EIF4G1 | 6.98847 | 4.22E-06 |
| HOXA1 | 5.626281 | 4.24E-06 |
| EHHADH | -6.77253 | 4.27E-06 |
| PSMD8 | 8.25955 | 4.28E-06 |
| COL10A1 | 8.564866 | 4.33E-06 |
| BGN | 8.781335 | 4.33E-06 |
| NDUFA4 | -9.73388 | 4.42E-06 |
| ME3 | -6.24314 | 4.50E-06 |
| RAF1 | -6.38488 | 4.54E-06 |
| EPHB2 | 6.977374 | 4.57E-06 |
| SLC14A1 | -4.33481 | 4.62E-06 |
| GMPR | -6.69612 | 4.62E-06 |
| NONO | 8.287977 | 4.62E-06 |
| FRK | -6.54978 | 4.65E-06 |
| SPTAN1 | 6.846071 | 4.65E-06 |
| CDH11 | 7.785428 | 4.69E-06 |
| SRI | 8.182126 | 4.69E-06 |
| ARHGDIG | -4.99632 | 4.76E-06 |
| CXCL1 | 9.485442 | 4.95E-06 |
| CENPE | 6.15413 | 5.04E-06 |
| SNTA1 | -5.61738 | 5.06E-06 |
| FOXM1 | 7.097636 | 5.31E-06 |
| C5AR1 | 7.603594 | 5.45E-06 |
| IMPDH1 | -6.45819 | 5.52E-06 |
| PSMB5 | 8.06795 | 5.53E-06 |
| PTPN4 | -5.11743 | 5.56E-06 |
| BMP2 | -7.52764 | 5.62E-06 |
| FHIT | -5.49184 | 5.67E-06 |
| TNFAIP6 | 7.743954 | 5.68E-06 |
| CENPF | 6.512193 | 5.70E-06 |
| HAS3 | -6.05644 | 5.91E-06 |
| GC | -8.76596 | 6.10E-06 |
| RPN1 | 8.485715 | 6.39E-06 |
| CXADR | -8.18655 | 6.43E-06 |
| 44626 | -6.65154 | 6.43E-06 |
| PCSK5 | 6.081685 | 6.54E-06 |
| ISG20 | -8.45484 | 6.97E-06 |
| DPT | -7.00802 | 7.04E-06 |
| SEC14L1 | -6.49243 | 7.08E-06 |
| PLA2G2A | 11.081 | 7.37E-06 |
| KEAP1 | 6.642207 | 7.40E-06 |
| CLINT1 | -7.51664 | 7.41E-06 |
| RQCD1 | 5.116705 | 7.41E-06 |
| PEA15 | 8.275172 | 7.41E-06 |
| GNAI2 | 7.055701 | 7.48E-06 |
| OASL | -7.65198 | 7.55E-06 |
| TRIP13 | 7.205932 | 7.55E-06 |
| RPL35 | 10.11242 | 7.56E-06 |
| CITED2 | -6.59696 | 7.62E-06 |
| FUT8 | -6.8554 | 8.02E-06 |
| BCKDHB | -6.12261 | 8.25E-06 |
| CAMK2D | -6.47849 | 8.30E-06 |
| STIP1 | 7.329435 | 8.43E-06 |
| PAICS | 7.775888 | 8.51E-06 |
| IL13RA2 | 5.492021 | 8.60E-06 |
| PROCR | 7.5292 | 8.70E-06 |
| DNMT1 | 7.728509 | 8.72E-06 |
| CDH3 | 8.559683 | 8.92E-06 |
| S100A4 | 10.49636 | 9.20E-06 |
| KIFC1 | 6.648368 | 9.21E-06 |
| NMI | 8.419511 | 9.35E-06 |
| KIAA0101 | 8.157671 | 9.36E-06 |
| PCSK7 | -6.40843 | 9.38E-06 |
| PTGS1 | -6.26697 | 9.38E-06 |
| KIAA0430 | -5.6703 | 9.61E-06 |
| ACVR2A | -5.78219 | 9.66E-06 |
| MSH2 | 6.581632 | 9.71E-06 |
| MMD | 6.622734 | 9.80E-06 |
| TAF11 | 5.853726 | 1.04E-05 |
| ADK | 6.506852 | 1.06E-05 |
| NAGLU | -4.99875 | 1.07E-05 |
| EPHB3 | -7.10823 | 1.11E-05 |
| PPP2R1B | -5.8926 | 1.11E-05 |
| XPO1 | 7.978392 | 1.11E-05 |
| PSMB7 | 7.938543 | 1.12E-05 |
| AQP1 | 8.627824 | 1.12E-05 |
| C1QBP | 8.946192 | 1.13E-05 |
| ADSL | 7.581677 | 1.14E-05 |
| KCNQ1 | -7.12829 | 1.17E-05 |
| TFAP2A | 6.535922 | 1.17E-05 |
| ATP5B | -9.40934 | 1.17E-05 |
| MDFI | 5.441425 | 1.17E-05 |
| CPT2 | -6.12962 | 1.18E-05 |
| PTS | -7.59131 | 1.19E-05 |
| GM2A | 6.734751 | 1.19E-05 |
| PPAT | 5.879626 | 1.19E-05 |
| PDK1 | -7.00742 | 1.20E-05 |
| ATP1B1 | -10.5376 | 1.20E-05 |
| HSD17B10 | 8.02547 | 1.20E-05 |
| DTYMK | 5.362798 | 1.22E-05 |
| GOLGA4 | -7.03826 | 1.24E-05 |
| MMP3 | 8.776859 | 1.24E-05 |
| CD55 | 9.863411 | 1.24E-05 |
| AGA | -6.41578 | 1.25E-05 |
| NCF2 | 6.90143 | 1.26E-05 |
| AHNAK | -7.29091 | 1.27E-05 |
| CDC27 | 5.752362 | 1.27E-05 |
| ADRM1 | 8.227767 | 1.28E-05 |
| RCAN2 | -7.54643 | 1.28E-05 |
| CDKN3 | 7.664181 | 1.29E-05 |
| PCOLCE | 7.616652 | 1.29E-05 |
| IL1R2 | -9.14406 | 1.32E-05 |
| CCT4 | 7.952878 | 1.34E-05 |
| MELK | 7.968908 | 1.34E-05 |
| GAPDH | 10.28821 | 1.37E-05 |
| PNPLA4 | -6.94877 | 1.39E-05 |
| FUS | 6.257411 | 1.40E-05 |
| KPNB1 | 7.915338 | 1.42E-05 |
| PLSCR1 | 8.096958 | 1.43E-05 |
| MAD1L1 | 6.296961 | 1.44E-05 |
| SSRP1 | 6.915662 | 1.47E-05 |
| ATR | 5.915114 | 1.47E-05 |
| TAGLN2 | -9.25666 | 1.48E-05 |
| COMMD1 | -6.97472 | 1.51E-05 |
| ITGB8 | 6.444328 | 1.56E-05 |
| DDX21 | 8.697847 | 1.56E-05 |
| RNASEL | -5.72076 | 1.57E-05 |
| MDK | 8.238953 | 1.57E-05 |
| SEC61B | -8.27266 | 1.57E-05 |
| THOP1 | 5.86017 | 1.62E-05 |
| SLC28A1 | -4.66995 | 1.63E-05 |
| CDK2 | 5.583123 | 1.68E-05 |
| CTSA | 8.663074 | 1.68E-05 |
| AP3B1 | -5.50984 | 1.76E-05 |
| MTHFD1 | 8.241695 | 1.76E-05 |
| ATF4 | -8.71056 | 1.78E-05 |
| PTDSS1 | 8.130307 | 1.78E-05 |
| GEM | 8.563456 | 1.78E-05 |
| S100A13 | 6.980695 | 1.80E-05 |
| PPIB | 9.298987 | 1.83E-05 |
| CPA3 | -8.38809 | 1.90E-05 |
| ABCG1 | -5.74237 | 1.90E-05 |
| DEFA4 | -4.87043 | 1.91E-05 |
| RNF103 | -7.29468 | 1.92E-05 |
| PIAS1 | -6.06567 | 1.92E-05 |
| DCTN2 | -5.87256 | 1.95E-05 |
| COL5A1 | 8.476659 | 1.98E-05 |
| PPFIA1 | 5.180329 | 1.98E-05 |
| CCNF | 5.010597 | 1.99E-05 |
| DNAJA1 | 8.70532 | 2.01E-05 |
| LAMA2 | -5.98115 | 2.06E-05 |
| STS | -6.53771 | 2.07E-05 |
| BARD1 | 5.568556 | 2.07E-05 |
| TRIP10 | 7.763731 | 2.08E-05 |
| SLC5A5 | -6.20737 | 2.12E-05 |
| IVD | -5.74214 | 2.12E-05 |
| PPT1 | 8.53974 | 2.12E-05 |
| THBS1 | 7.076371 | 2.13E-05 |
| MSLN | 8.325696 | 2.17E-05 |
| GPNMB | 9.153314 | 2.18E-05 |
| RGS16 | 5.433646 | 2.19E-05 |
| NR2F6 | -6.17468 | 2.21E-05 |
| CA4 | -5.33848 | 2.22E-05 |
| E2F3 | 6.105194 | 2.25E-05 |
| PRMT5 | 6.91404 | 2.25E-05 |
| LMNB1 | 7.453774 | 2.25E-05 |
| YWHAB | 8.56172 | 2.25E-05 |
| GNPDA1 | 6.764903 | 2.26E-05 |
| BST2 | 8.794175 | 2.26E-05 |
| SPCS2 | -8.05578 | 2.26E-05 |
| SCRIB | 7.004628 | 2.27E-05 |
| GAMT | -5.92128 | 2.29E-05 |
| NR2F2 | -7.91167 | 2.30E-05 |
| TIMP2 | 8.190082 | 2.31E-05 |
| KLC1 | 5.680673 | 2.33E-05 |
| PTN | -5.84381 | 2.43E-05 |
| ZNF267 | 6.149556 | 2.49E-05 |
| PTPRO | -10.1022 | 2.49E-05 |
| PRKX | 6.411137 | 2.54E-05 |
| TYMS | 8.139381 | 2.54E-05 |
| IL24 | 5.458674 | 2.56E-05 |
| ACADS | -5.93963 | 2.57E-05 |
| TMSB4X | -11.8799 | 2.57E-05 |
| CD27 | -7.54923 | 2.59E-05 |
| CDS1 | -7.48457 | 2.60E-05 |
| TCOF1 | 4.899013 | 2.64E-05 |
| CA8 | -4.52767 | 2.69E-05 |
| H2AFX | 6.562743 | 2.69E-05 |
| PSMD12 | 6.540019 | 2.71E-05 |
| F12 | 6.211377 | 2.73E-05 |
| CDH2 | -6.51209 | 2.77E-05 |
| ITGB7 | -5.95353 | 2.78E-05 |
| CCT2 | 8.475275 | 2.78E-05 |
| ATP6V1C1 | 6.194382 | 2.79E-05 |
| PRPF18 | -5.22185 | 2.82E-05 |
| FBN1 | 7.808383 | 2.84E-05 |
| EEF1A2 | -6.53918 | 2.97E-05 |
| SELE | 5.591388 | 2.98E-05 |
| MSR1 | 4.51036 | 3.02E-05 |
| RPL34 | -11.0716 | 3.04E-05 |
| COX10 | -6.55319 | 3.05E-05 |
| SSTR2 | -4.29254 | 3.11E-05 |
| ESR2 | -3.2304 | 3.27E-05 |
| SHC1 | 7.229067 | 3.43E-05 |
| DNASE1L3 | -5.93441 | 3.45E-05 |
| ANXA2 | 9.984249 | 3.51E-05 |
| IL15RA | 6.107441 | 3.51E-05 |
| ESPL1 | 6.307779 | 3.51E-05 |
| ALG3 | 7.15296 | 3.53E-05 |
| GIPR | -4.46407 | 3.58E-05 |
| SKP2 | 6.763738 | 3.58E-05 |
| C1R | 8.485753 | 3.61E-05 |
| MAPK3 | -7.08389 | 3.66E-05 |
| RPL37A | -10.377 | 3.66E-05 |
| PBX1 | -7.23823 | 3.71E-05 |
| PPP1R8 | 6.352896 | 3.71E-05 |
| RAB1A | -8.07826 | 3.72E-05 |
| CSTF2 | 5.847642 | 3.75E-05 |
| COX7A2 | -10.4151 | 3.77E-05 |
| B2M | -11.3499 | 3.78E-05 |
| SALL1 | -4.44986 | 3.81E-05 |
| FOXA1 | -5.94486 | 3.83E-05 |
| CDC25A | 4.593532 | 3.96E-05 |
| CCNB1 | 7.795745 | 4.02E-05 |
| TSPAN31 | -7.05466 | 4.13E-05 |
| F13A1 | -8.37503 | 4.21E-05 |
| GCNT1 | -7.54328 | 4.21E-05 |
| YIPF6 | -6.36937 | 4.33E-05 |
| CBFB | 7.009739 | 4.33E-05 |
| PSMC5 | 7.571216 | 4.38E-05 |
| DCK | 7.280351 | 4.43E-05 |
| APOC1 | 8.970766 | 4.43E-05 |
| RCN2 | 8.089736 | 4.58E-05 |
| RNF10 | -6.63431 | 4.58E-05 |
| PTEN | -5.99289 | 4.61E-05 |
| IMPDH2 | 8.637719 | 4.68E-05 |
| ITGB5 | 6.610148 | 4.81E-05 |
| LMNA | 7.64137 | 4.85E-05 |
| PLOD1 | 7.296065 | 4.99E-05 |
| MCM7 | 8.016807 | 5.03E-05 |
| SMPDL3A | -7.58175 | 5.09E-05 |
| CTSK | 8.586495 | 5.11E-05 |
| HOXB3 | 6.789151 | 5.15E-05 |
| GGH | 8.635057 | 5.17E-05 |
| CLIC1 | 9.369897 | 5.19E-05 |
| EDEM1 | -6.71115 | 5.38E-05 |
| LIG1 | 5.852489 | 5.40E-05 |
| WASF3 | -6.56046 | 5.49E-05 |
| LRBA | -6.84242 | 5.50E-05 |
| PDK4 | -7.74166 | 5.53E-05 |
| COL6A2 | 8.228854 | 5.65E-05 |
| HMGB1 | 8.870421 | 5.65E-05 |
| CTBS | -6.48067 | 5.82E-05 |
| LSS | -5.85527 | 5.91E-05 |
| TUBGCP3 | 5.772317 | 5.98E-05 |
| PRPH2 | -5.27962 | 6.09E-05 |
| ZNF239 | 5.716883 | 6.33E-05 |
| IRAK1 | 7.704717 | 6.43E-05 |
| SERPING1 | 8.475459 | 6.54E-05 |
| BCL6 | 6.100947 | 6.57E-05 |
| ACYP1 | 5.863205 | 6.64E-05 |
| SFXN3 | 6.256584 | 6.82E-05 |
| TGFBR1 | 6.306522 | 6.88E-05 |
| GPSM2 | 5.969648 | 7.07E-05 |
| IER2 | 8.532784 | 7.09E-05 |
| FABP3 | -5.55196 | 7.10E-05 |
| ELK4 | 5.400754 | 7.10E-05 |
| PSMB2 | 7.342164 | 7.14E-05 |
| OSBP | -5.70475 | 7.41E-05 |
| FCGR2A | 6.484551 | 7.41E-05 |
| PON2 | 8.450364 | 7.59E-05 |
| DDX10 | 5.329042 | 7.60E-05 |
| MNDA | 7.029799 | 7.63E-05 |
| RRP1B | 7.151406 | 7.64E-05 |
| E2F4 | -6.72905 | 7.65E-05 |
| TFDP2 | 5.437635 | 7.67E-05 |
| HUWE1 | -9.43837 | 7.68E-05 |
| PIM2 | -6.96121 | 7.68E-05 |
| PUM2 | -6.93231 | 7.68E-05 |
| DNM2 | -5.20959 | 7.68E-05 |
| PRIM2 | 5.766592 | 7.68E-05 |
| ERF | 5.05296 | 7.73E-05 |
| PWP1 | 6.827791 | 7.88E-05 |
| TNFSF4 | 5.55163 | 8.06E-05 |
| GRK5 | -6.27688 | 8.11E-05 |
| CHKA | -6.00835 | 8.11E-05 |
| SIRPA | 5.578682 | 8.20E-05 |
| TRADD | -5.69509 | 8.26E-05 |
| GPC1 | 6.00591 | 8.36E-05 |
| FCER1G | 8.410286 | 8.38E-05 |
| PFN2 | 8.388087 | 8.55E-05 |
| FUT2 | -7.15748 | 8.56E-05 |
| SUOX | -5.73525 | 8.56E-05 |
| ODC1 | 9.190016 | 8.58E-05 |
| POFUT1 | 5.434136 | 8.77E-05 |
| VLDLR | -6.38675 | 9.08E-05 |
| CLOCK | -6.30117 | 9.15E-05 |
| ACVR1B | -5.71538 | 9.42E-05 |
| UNG | 7.144787 | 9.52E-05 |
| PSMD14 | 8.175152 | 9.61E-05 |
| U2AF1 | 6.430845 | 9.68E-05 |
| F2R | 6.640271 | 9.79E-05 |
| GABRB3 | -5.39745 | 9.91E-05 |
| PTGES3 | 9.224452 | 0.000101 |
| CD63 | -9.38089 | 0.000101 |
| CEACAM6 | 10.8208 | 0.000101 |
| COX6A2 | -4.61911 | 0.000102 |
| PSMC3 | 7.203864 | 0.000103 |
| TIMM17A | 6.661537 | 0.000103 |
| PIK3C3 | -4.07797 | 0.000103 |
| P2RY6 | 4.893033 | 0.000105 |
| CPB1 | -6.76406 | 0.000105 |
| MMP1 | 9.650149 | 0.000106 |
| ARHGAP11A | 4.169393 | 0.000108 |
| PMPCA | 7.1587 | 0.000109 |
| DGUOK | 5.935533 | 0.000109 |
| POLRMT | 4.993966 | 0.00011 |
| GRIA4 | -4.76278 | 0.000111 |
| DPF2 | 6.160005 | 0.000111 |
| TPR | 6.057252 | 0.000111 |
| CCNA2 | 6.874051 | 0.000112 |
| FSTL1 | 8.395183 | 0.000112 |
| ABI2 | 5.062847 | 0.000113 |
| SERPINE1 | 7.159161 | 0.000114 |
| SCARB2 | -7.21563 | 0.000114 |
| TOMM34 | 6.878153 | 0.000114 |
| GUCY1A3 | 7.560388 | 0.000116 |
| KCNJ8 | 5.43036 | 0.000116 |
| SLC25A3 | -8.30295 | 0.000118 |
| RPL7L1 | 8.230967 | 0.000121 |
| TNFAIP2 | 7.344322 | 0.000122 |
| RPS14 | -9.00298 | 0.000123 |
| FGFR4 | 5.544726 | 0.000126 |
| IGLL1 | -4.94855 | 0.000126 |
| IFNGR1 | 8.061727 | 0.000127 |
| MAP3K10 | -3.66191 | 0.000127 |
| CDH13 | 4.648069 | 0.000128 |
| ITPR1 | -5.86081 | 0.000129 |
| TGM2 | 7.126431 | 0.000129 |
| PTGS2 | 7.425652 | 0.000129 |
| RPA3 | 8.177426 | 0.000129 |
| ZKSCAN3 | -4.18426 | 0.00013 |
| FGF13 | -6.23475 | 0.000131 |
| VCAN | 8.722876 | 0.000132 |
| ITGA2 | 8.115626 | 0.000134 |
| SRPR | -7.38619 | 0.000135 |
| PTCH1 | -6.17998 | 0.000135 |
| PLD3 | 7.289539 | 0.000136 |
| USP14 | 7.220623 | 0.000137 |
| PTPN5 | -3.55648 | 0.000138 |
| TRIM15 | 6.637861 | 0.00014 |
| AVP | -4.08891 | 0.000141 |
| SDS | 4.993906 | 0.00015 |
| KIF2A | 5.959254 | 0.000153 |
| MYD88 | -7.95163 | 0.000154 |
| RARRES1 | 9.096656 | 0.000155 |
| ADH1B | -7.75998 | 0.000155 |
| ATXN2L | 4.21124 | 0.000156 |
| DPYSL2 | 8.381502 | 0.000157 |
| SMARCA4 | 5.994715 | 0.000158 |
| EMR1 | -5.69566 | 0.00016 |
| SPOCK1 | 6.85696 | 0.00016 |
| PTPN21 | -4.28772 | 0.000162 |
| BCAT1 | 6.10362 | 0.000163 |
| LMNB2 | 6.884129 | 0.000164 |
| GCH1 | 7.179775 | 0.000168 |
| RIPK1 | -5.08396 | 0.00017 |
| KIAA0232 | -6.01434 | 0.00017 |
| MAOA | -8.35105 | 0.00017 |
| DOCK2 | -5.50708 | 0.000171 |
| PAK2 | 5.581468 | 0.000171 |
| SERPINB5 | 9.354082 | 0.000171 |
| FZD2 | 5.813613 | 0.000174 |
| HMBS | 6.24716 | 0.000174 |
| PAFAH1B1 | -5.84641 | 0.000175 |
| HEXB | 8.484913 | 0.000175 |
| CALD1 | 7.391405 | 0.000176 |
| AFM | -3.95261 | 0.000178 |
| STOM | 7.61514 | 0.000179 |
| WNT2 | 6.012392 | 0.000181 |
| SLC10A3 | 6.03075 | 0.000182 |
| HMGB2 | 8.326901 | 0.000183 |
| IFIT3 | 8.082221 | 0.000183 |
| FOXD1 | 6.236062 | 0.000184 |
| EXOSC2 | 4.58917 | 0.000184 |
| GAB1 | -4.70758 | 0.000186 |
| GUCA2B | -7.73439 | 0.000186 |
| PHB | 7.431977 | 0.000195 |
| ITGA1 | 6.070209 | 0.000196 |
| GNAZ | -5.32906 | 0.000197 |
| SRPX | -7.78859 | 0.000198 |
| CTAGE5 | -5.49689 | 0.000198 |
| ERBB4 | -3.97331 | 0.000199 |
| SNTB1 | -5.35223 | 0.000201 |
| ICAM1 | 6.935126 | 0.000201 |
| PNO1 | 6.290474 | 0.000201 |
| WNT5A | 7.440445 | 0.000202 |
| UBE2A | 6.957915 | 0.000206 |
| TMPO | 6.586703 | 0.000207 |
| GJB2 | 8.742038 | 0.00021 |
| MAP2K4 | -5.05007 | 0.000216 |
| STAB1 | -5.40553 | 0.000218 |
| RAB30 | -5.31434 | 0.000219 |
| DPM1 | 8.765931 | 0.00022 |
| LTK | -4.30147 | 0.00022 |
| KAL1 | 5.087443 | 0.000222 |
| SLC10A1 | -6.34318 | 0.000222 |
| HSPH1 | 7.555966 | 0.000226 |
| RBM6 | -6.73935 | 0.000227 |
| FSCN1 | 6.686908 | 0.000227 |
| NUBP1 | -6.08645 | 0.000228 |
| SAC3D1 | 6.819373 | 0.000233 |
| BTG1 | -8.75192 | 0.000233 |
| CAT | -7.77873 | 0.000234 |
| ENPP2 | -7.85846 | 0.000236 |
| HMGN4 | 7.810066 | 0.000237 |
| GATA4 | -5.79919 | 0.000237 |
| CYP27A1 | 6.439962 | 0.000238 |
| CTNND2 | -3.51586 | 0.000239 |
| RPS24 | -9.83116 | 0.000239 |
| TRIM28 | 8.775075 | 0.000241 |
| KPNA4 | 6.934044 | 0.000242 |
| H2AFZ | 9.090561 | 0.000243 |
| NCAPD2 | 6.324914 | 0.000245 |
| NFIL3 | 7.284312 | 0.000247 |
| IL11RA | -4.75471 | 0.000247 |
| DDX18 | 7.233332 | 0.000247 |
| RABEPK | 5.357135 | 0.00025 |
| SUMO1 | 7.215496 | 0.000255 |
| PLA2G7 | 7.787883 | 0.000257 |
| NCAPH | 5.771777 | 0.00026 |
| ANK3 | -5.70123 | 0.000262 |
| ACSL1 | -7.48311 | 0.000266 |
| NINJ1 | 6.621207 | 0.000272 |
| PLAUR | 8.081149 | 0.000273 |
| ATP1B2 | -4.32576 | 0.000276 |
| MCM6 | 7.198093 | 0.000276 |
| GOLGB1 | -6.5205 | 0.000281 |
| BRCA1 | 5.361142 | 0.000283 |
| PLA2G4A | 7.328232 | 0.000283 |
| ELK3 | 6.640292 | 0.000283 |
| GSTZ1 | -5.36811 | 0.000284 |
| CCND2 | 7.896199 | 0.000284 |
| TNFRSF25 | 4.271643 | 0.000286 |
| GNAQ | -7.0219 | 0.000286 |
| ALG13 | -5.58753 | 0.000287 |
| PTPRA | 5.244072 | 0.000292 |
| HLX | 4.757832 | 0.000292 |
| DAPK1 | -6.45519 | 0.000295 |
| TDRD9 | -4.04526 | 0.000296 |
| STAT5A | 6.004909 | 0.000298 |
| SLC2A3 | 7.053202 | 0.0003 |
| HNRNPU | 7.13842 | 0.000302 |
| FLNA | 8.376784 | 0.000302 |
| TNFRSF8 | -4.31272 | 0.000304 |
| FUBP1 | 5.206608 | 0.000309 |
| LAMC2 | 7.524471 | 0.00031 |
| HSPA4 | 6.146699 | 0.000315 |
| LILRB4 | 4.841352 | 0.000315 |
| ABCA2 | -4.58407 | 0.000317 |
| RAD54L | 5.177167 | 0.000318 |
| ADRA2B | -4.08681 | 0.000318 |
| GRN | 8.211864 | 0.00032 |
| SLC6A11 | -3.33235 | 0.000321 |
| MXI1 | -7.24209 | 0.000326 |
| LRRC32 | 6.369269 | 0.000327 |
| RPS6 | -9.7619 | 0.000332 |
| SNRPC | 7.348098 | 0.000333 |
| SAT1 | -9.2429 | 0.000334 |
| NDUFV2 | -8.02156 | 0.000334 |
| MCM3 | 8.024126 | 0.000334 |
| PLCG2 | -5.66365 | 0.000335 |
| IGFBP4 | 8.883187 | 0.000336 |
| CAMK2G | 4.937646 | 0.000337 |
| RB1 | 6.646989 | 0.000341 |
| RBL1 | 3.890302 | 0.000342 |
| HBEGF | -7.10265 | 0.000342 |
| MMP14 | 6.066296 | 0.000347 |
| CENPI | 3.328871 | 0.000355 |
| IL10RB | -7.24229 | 0.000361 |
| NAP1L1 | 8.146353 | 0.000362 |
| CLDN5 | -6.03438 | 0.000364 |
| LEPROT | -6.84837 | 0.000364 |
| SNRK | -5.68516 | 0.000367 |
| ATP2A2 | 7.080418 | 0.000368 |
| CPM | -5.77992 | 0.000372 |
| ZNF274 | -5.94919 | 0.000377 |
| YBX1 | 8.681863 | 0.000383 |
| RECQL | 6.538677 | 0.000384 |
| ADORA2B | 6.81356 | 0.000384 |
| UQCRC1 | -8.25967 | 0.000389 |
| TKT | 7.987697 | 0.000394 |
| RFC4 | 7.388038 | 0.000395 |
| ARHGEF16 | -6.1306 | 0.000397 |
| KLF3 | -6.86376 | 0.0004 |
| IGF2BP3 | 6.166523 | 0.000404 |
| OS9 | -7.42426 | 0.000407 |
| TBC1D22A | -5.50427 | 0.000408 |
| RPN2 | 9.209375 | 0.000408 |
| HSF1 | 5.447451 | 0.000409 |
| ANXA6 | 6.35989 | 0.000409 |
| PDE1C | -3.73513 | 0.000411 |
| RPS18 | -11.128 | 0.000413 |
| ZBTB16 | -6.93282 | 0.000415 |
| SNRPB2 | 7.488891 | 0.000418 |
| NPAS1 | -3.73691 | 0.000419 |
| SMC1A | 6.550357 | 0.000422 |
| C2 | 6.356969 | 0.000423 |
| DPYSL3 | 6.974584 | 0.000423 |
| SCTR | -3.84898 | 0.000427 |
| DDX17 | -7.36959 | 0.000434 |
| LOX | 5.775788 | 0.00044 |
| DEFA5 | -10.5802 | 0.000442 |
| PKMYT1 | 5.882404 | 0.000442 |
| DEK | 8.367728 | 0.000442 |
| MTHFD2 | 7.312601 | 0.000445 |
| POU2F2 | -4.64808 | 0.000447 |
| KHSRP | 6.200066 | 0.000456 |
| CCR1 | 6.209721 | 0.000461 |
| RFC2 | 5.642832 | 0.000472 |
| PLAU | 8.517742 | 0.000472 |
| TDO2 | 5.097507 | 0.000476 |
| FANCA | 4.224915 | 0.000481 |
| CLCNKB | -4.30446 | 0.000482 |
| KRT9 | -4.98911 | 0.000484 |
| AP2M1 | 7.999484 | 0.000484 |
| USP13 | 5.106348 | 0.000485 |
| ATP6V1B2 | 6.987873 | 0.000487 |
| ALDOB | -9.29555 | 0.000488 |
| TM4SF1 | 8.746745 | 0.000494 |
| C6 | -6.40562 | 0.0005 |
| MMP15 | -4.9675 | 0.000514 |
| HMGN1 | 8.727541 | 0.000514 |
| POSTN | -8.64651 | 0.000526 |
| MRPS12 | 5.828166 | 0.000534 |
| NAB2 | 4.49609 | 0.000536 |
| TSPAN8 | -10.808 | 0.000539 |
| PGM1 | -7.85253 | 0.000539 |
| OPRM1 | -3.42572 | 0.000541 |
| CCNI | -7.90327 | 0.000551 |
| NR1D2 | -6.64774 | 0.000555 |
| BCKDHA | -6.49471 | 0.000568 |
| PPARD | -5.84915 | 0.000571 |
| TNFSF9 | 6.186395 | 0.000574 |
| PNN | -7.12744 | 0.000578 |
| RPL41 | -11.7923 | 0.000581 |
| UBE2D3 | -6.72712 | 0.000581 |
| CHRNA2 | -4.62129 | 0.000581 |
| ILF2 | 8.195771 | 0.000586 |
| MAPRE3 | -3.1091 | 0.000588 |
| ZNF169 | -5.69819 | 0.000592 |
| MAPRE2 | -5.53955 | 0.000594 |
| NR2C1 | 4.26089 | 0.000594 |
| CASP7 | -7.61453 | 0.000606 |
| LUM | 9.378224 | 0.000607 |
| VAT1 | 7.40711 | 0.000611 |
| RPS28 | -8.66788 | 0.000621 |
| ZNF24 | -5.14496 | 0.000623 |
| CD44 | 7.167566 | 0.000628 |
| EIF2AK2 | 6.255354 | 0.000632 |
| CD164 | -8.97461 | 0.000634 |
| HMMR | 6.895577 | 0.000634 |
| SF3A3 | 6.214382 | 0.000635 |
| ILK | 7.228957 | 0.000637 |
| TFDP1 | 6.34462 | 0.00064 |
| PNLIPRP2 | -7.67926 | 0.000646 |
| GP1BA | -5.24205 | 0.000646 |
| PIGB | -4.93193 | 0.000648 |
| POLR2G | 7.349844 | 0.000652 |
| NFKBIE | 5.738234 | 0.000654 |
| ANXA5 | 8.58695 | 0.000655 |
| OLR1 | 5.556901 | 0.000661 |
| COL6A1 | 6.782109 | 0.000661 |
| SLC7A6 | 5.064447 | 0.000662 |
| PPP2R4 | 6.175576 | 0.000664 |
| NKRF | 5.090381 | 0.000676 |
| HAPLN1 | -5.92311 | 0.00068 |
| CTSG | -5.50191 | 0.000686 |
| GDF15 | 7.732039 | 0.000687 |
| COL15A1 | 8.628874 | 0.000687 |
| RING1 | -5.64791 | 0.000687 |
| CACNB3 | 5.004923 | 0.000704 |
| ILVBL | -5.1655 | 0.000714 |
| MMP9 | 8.662323 | 0.000717 |
| PDGFRB | 6.469685 | 0.000726 |
| FOXF2 | -6.79608 | 0.000726 |
| PCCA | -6.47275 | 0.000726 |
| ZNF91 | -6.60084 | 0.000728 |
| BCL2A1 | 7.869123 | 0.000728 |
| SOX9 | 9.061104 | 0.000728 |
| CDA | 6.458452 | 0.000733 |
| PDE3A | 4.916769 | 0.000735 |
| IGHMBP2 | -4.24596 | 0.000744 |
| SRM | 6.718002 | 0.000761 |
| STAT3 | 6.90561 | 0.000761 |
| NOTCH3 | 6.237769 | 0.000764 |
| RALGDS | 6.620419 | 0.000769 |
| RBM3 | 7.089272 | 0.000784 |
| TPH1 | -5.59821 | 0.000801 |
| MTMR11 | 6.948643 | 0.000812 |
| ESRRA | -5.68908 | 0.00083 |
| TRIO | 4.359504 | 0.00083 |
| LSR | 8.537902 | 0.00083 |
| ERH | 8.602609 | 0.00083 |
| PSMB9 | 8.815189 | 0.000834 |
| ABCE1 | 7.036683 | 0.000838 |
| AHR | 7.974303 | 0.000839 |
| ETV2 | -4.17794 | 0.000844 |
| CPE | -7.71394 | 0.000846 |
| PDGFRL | 5.227625 | 0.000852 |
| FAM3A | -5.22729 | 0.000858 |
| RBM10 | 6.002047 | 0.00086 |
| MAGEA12 | 7.863538 | 0.000868 |
| BAD | -4.52111 | 0.000871 |
| MYB | 6.422642 | 0.000875 |
| EEF2 | -9.45324 | 0.000876 |
| HSD11B1 | 5.934763 | 0.000881 |
| TTK | 6.969613 | 0.000884 |
| DUSP5 | -6.86575 | 0.000884 |
| MAN2C1 | -4.14081 | 0.00091 |
| ARHGEF2 | -5.40645 | 0.000919 |
| GPM6A | -3.85717 | 0.000923 |
| SLC35A2 | 5.842201 | 0.000928 |
| ZNF134 | -4.71667 | 0.000933 |
| TNFRSF11B | 6.090526 | 0.000933 |
| MRE11A | 4.308116 | 0.000935 |
| VAMP2 | -6.21911 | 0.000938 |
| LPAR1 | -5.5108 | 0.000938 |
| PRCC | 6.046624 | 0.000941 |
| SRF | 4.673276 | 0.000952 |
| PTPRS | -4.64642 | 0.000961 |
| CFD | -8.72536 | 0.000963 |
| C1S | 8.548213 | 0.000964 |
| VAC14 | 4.400512 | 0.00097 |
| GNL2 | 6.45826 | 0.000984 |
| MCF2 | -2.75481 | 0.000986 |
| BZW1 | 7.429747 | 0.000986 |
| ITIH4 | -3.71048 | 0.001 |
| RBMS2 | 4.352347 | 0.001036 |
| LAMA4 | 5.225331 | 0.001063 |
| TAP1 | 8.910265 | 0.001064 |
| CSNK2B | 8.286348 | 0.001064 |
| SCN4A | -4.20629 | 0.001066 |
| RPA1 | 6.045995 | 0.001077 |
| SART3 | 4.737898 | 0.001078 |
| MYH9 | 7.414433 | 0.001078 |
| SEMA4D | -5.58461 | 0.00108 |
| EXT1 | 5.921562 | 0.001087 |
| EFTUD2 | 6.683387 | 0.001102 |
| NPM1 | 8.556004 | 0.001102 |
| MUC5B | 7.54381 | 0.001108 |
| ATP7A | -5.42148 | 0.001108 |
| GTF2F2 | 5.820071 | 0.001112 |
| SERPINA5 | -6.69638 | 0.00112 |
| PTPN18 | -5.38908 | 0.00112 |
| ST5 | -6.34529 | 0.001121 |
| FHL1 | -8.23857 | 0.001122 |
| DGCR5 | -3.16066 | 0.001127 |
| FDXR | 5.806248 | 0.001127 |
| DNM1L | 6.021901 | 0.001131 |
| ARCN1 | -7.40393 | 0.001143 |
| STAT1 | 8.434048 | 0.001143 |
| KRT7 | 7.253432 | 0.001145 |
| RAB3GAP1 | -5.74694 | 0.001155 |
| CRABP2 | 7.40451 | 0.001168 |
| FKBP1B | -7.56707 | 0.001168 |
| TUBG1 | 6.762303 | 0.001185 |
| PDE2A | -5.25813 | 0.001191 |
| TAZ | -4.99495 | 0.001204 |
| HSD17B1 | 4.729474 | 0.001209 |
| UNC119 | 5.124039 | 0.001215 |
| MDM4 | -5.74543 | 0.001229 |
| P2RX1 | -4.5158 | 0.001231 |
| NUDT1 | 5.348003 | 0.001235 |
| FKBP2 | -6.9896 | 0.001252 |
| BRCA2 | 4.67017 | 0.001253 |
| AGPS | 6.031838 | 0.001255 |
| DLG1 | -4.93161 | 0.001276 |
| UBC | -9.67935 | 0.001276 |
| RPL36AL | -9.19985 | 0.001281 |
| ADRA1D | -2.74514 | 0.001286 |
| RGN | -6.56745 | 0.001293 |
| OR1E1 | -4.93146 | 0.001298 |
| PABPC1 | 8.808846 | 0.001298 |
| LYZ | -10.7574 | 0.001304 |
| PGGT1B | -5.81384 | 0.001306 |
| SCAMP1 | -5.65157 | 0.001324 |
| TSFM | 4.981615 | 0.001324 |
| ST3GAL2 | 4.112963 | 0.001325 |
| MST1R | -6.90902 | 0.001326 |
| ARHGEF5 | -5.82709 | 0.001326 |
| PFKP | 7.094316 | 0.001326 |
| CCKAR | -5.32095 | 0.001359 |
| CASR | -3.30242 | 0.001366 |
| BNIP1 | -3.9125 | 0.001369 |
| SPTBN1 | 6.630087 | 0.001369 |
| TAF12 | 5.469151 | 0.001371 |
| PTPN14 | 4.939252 | 0.001384 |
| TMEM41B | -6.82053 | 0.001393 |
| GPR176 | 4.336718 | 0.001393 |
| RGR | -3.8736 | 0.001401 |
| LRMP | -6.48543 | 0.001401 |
| PTK2B | -4.96358 | 0.001403 |
| FAM3C | -7.18432 | 0.001406 |
| IL11 | 4.242877 | 0.001406 |
| TFPI2 | 5.002521 | 0.001421 |
| PTPN3 | -5.21218 | 0.001429 |
| MEP1B | -7.21805 | 0.001443 |
| RAD51 | 5.093723 | 0.001443 |
| TFAP2C | 5.247283 | 0.001449 |
| ANXA1 | 8.571133 | 0.001449 |
| SLC1A3 | 5.536424 | 0.001468 |
| PLCL1 | -4.176 | 0.001496 |
| AEBP1 | 8.10932 | 0.001517 |
| NUP160 | 5.661018 | 0.001529 |
| DDB1 | 7.057172 | 0.00153 |
| ATP5C1 | -8.79721 | 0.001531 |
| APOBEC3B | 7.342426 | 0.001535 |
| PRKAB1 | -5.70731 | 0.001546 |
| HLA-DOB | -5.56013 | 0.001554 |
| CYCS | -8.1738 | 0.001618 |
| RAP1A | -6.74252 | 0.001622 |
| TGM3 | -4.34764 | 0.001628 |
| FCAR | -2.84291 | 0.001633 |
| AKAP13 | -5.80895 | 0.001637 |
| CNR2 | -5.64933 | 0.001642 |
| GSN | -6.47637 | 0.001651 |
| TSC1 | -5.87232 | 0.001664 |
| PDGFRA | -6.70807 | 0.001672 |
| SHMT2 | 6.808362 | 0.00168 |
| HTR6 | -3.97079 | 0.001681 |
| CD24 | -9.83004 | 0.001688 |
| HRK | -3.25881 | 0.00169 |
| PDE6A | -3.39775 | 0.001701 |
| HABP2 | -7.00563 | 0.001705 |
| CNGB1 | -3.30657 | 0.001719 |
| ATP12A | -4.0292 | 0.001722 |
| GAD1 | 4.797745 | 0.001723 |
| CCL8 | 6.51796 | 0.001725 |
| BECN1 | -6.16187 | 0.001735 |
| ACOX1 | -5.90959 | 0.001742 |
| ADCY2 | -3.76487 | 0.001751 |
| EFR3A | 7.47536 | 0.001759 |
| ACLY | 6.786322 | 0.001766 |
| HNRNPA3P1 | -3.67833 | 0.001772 |
| SKI | 7.006808 | 0.001772 |
| KCNB1 | -3.64989 | 0.001782 |
| BCAT2 | -6.45029 | 0.001811 |
| COX5B | -8.41786 | 0.001818 |
| NUP205 | 5.882459 | 0.001823 |
| KRT6B | 8.236768 | 0.001825 |
| MAGEA1 | 5.667485 | 0.001835 |
| MICB | 6.61012 | 0.001861 |
| PXDN | 6.72733 | 0.001861 |
| CXCL10 | 9.216369 | 0.001882 |
| CCT6B | -4.65275 | 0.001885 |
| ZNF146 | 6.72514 | 0.001885 |
| SLC25A16 | -5.51048 | 0.001886 |
| CHAF1B | 4.857873 | 0.001893 |
| PARP1 | 7.280716 | 0.001906 |
| PI3 | 10.09181 | 0.001914 |
| RHD | -3.87851 | 0.001917 |
| IL16 | -5.52406 | 0.001918 |
| NFRKB | 3.389372 | 0.001923 |
| MORF4L2 | 7.742891 | 0.001952 |
| RPS27 | -10.3109 | 0.001964 |
| HRAS | 6.111719 | 0.00198 |
| SNRPA | 7.004387 | 0.00199 |
| TP53 | 6.7343 | 0.002002 |
| CBLB | -5.30295 | 0.002013 |
| BLK | -4.34669 | 0.002031 |
| FPGS | 5.315438 | 0.002037 |
| HIST1H3B | 3.731495 | 0.002039 |
| TPM2 | 7.666965 | 0.00206 |
| ADCY1 | -3.08368 | 0.002114 |
| MATN3 | 5.48509 | 0.002171 |
| MCM5 | 6.295439 | 0.002192 |
| IFIT2 | 6.632076 | 0.00222 |
| IFI16 | 8.218058 | 0.002221 |
| EED | 5.689782 | 0.002228 |
| CCNC | -6.98342 | 0.002243 |
| TFF3 | 9.72603 | 0.002247 |
| CYP2E1 | -4.79251 | 0.002259 |
| KIF11 | 6.606527 | 0.002259 |
| CGREF1 | 5.146608 | 0.00227 |
| PTAFR | 5.461058 | 0.00227 |
| RBP4 | 7.315497 | 0.00227 |
| HTR3A | -3.60691 | 0.002278 |
| CYP4A11 | -4.43209 | 0.002289 |
| PYGB | 7.585744 | 0.002291 |
| TRAF3 | 4.72495 | 0.002294 |
| PTPRD | -3.79334 | 0.002301 |
| LASP1 | -8.48811 | 0.002302 |
| DCTD | -6.11353 | 0.002302 |
| GALNT1 | 7.242079 | 0.002304 |
| RPL12 | 9.960833 | 0.002329 |
| PTK6 | -6.31945 | 0.002366 |
| SLC6A2 | -2.40677 | 0.002385 |
| SIM2 | -4.81264 | 0.002388 |
| TBXA2R | -3.87633 | 0.002391 |
| SLC18A1 | -4.56936 | 0.002395 |
| MFSD10 | 5.686767 | 0.002403 |
| MIPEP | 5.942454 | 0.002403 |
| SEC62 | -7.65267 | 0.002415 |
| SOAT1 | -5.98112 | 0.002432 |
| MSMB | -9.71288 | 0.002442 |
| MCC | -5.37409 | 0.002448 |
| COMP | 6.247139 | 0.002454 |
| S100A9 | 8.131732 | 0.002467 |
| SDHA | -7.47042 | 0.002475 |
| MTA1 | 5.299049 | 0.00248 |
| CDX1 | 8.02312 | 0.002493 |
| ITPR3 | -6.26514 | 0.002495 |
| WWP2 | -4.35253 | 0.002529 |
| ACADSB | -5.71284 | 0.002543 |
| NCOA1 | -5.73342 | 0.002557 |
| GAL | 5.573756 | 0.002565 |
| GATA2 | -4.39337 | 0.002566 |
| PLCD1 | -5.65415 | 0.002573 |
| NR0B1 | -3.46141 | 0.002614 |
| EIF2S1 | 6.305767 | 0.002614 |
| GSTM5 | -5.4636 | 0.002621 |
| NFIX | -5.49417 | 0.002643 |
| KCNJ2 | 6.301491 | 0.002646 |
| ZNF33A | -4.77825 | 0.002666 |
| APCS | -4.36988 | 0.002668 |
| SCARB1 | 5.477874 | 0.002689 |
| NTRK3 | -3.71044 | 0.002692 |
| VEGFC | 4.866796 | 0.002693 |
| EPHB1 | 4.898139 | 0.002717 |
| PCBD1 | 7.188778 | 0.002722 |
| PDAP1 | 6.413638 | 0.002722 |
| PPY | -4.43967 | 0.00274 |
| PPID | 6.308276 | 0.002743 |
| GSTP1 | 8.940209 | 0.002751 |
| MTMR1 | 4.942028 | 0.002765 |
| FTH1 | 9.012598 | 0.002765 |
| CD14 | 7.752213 | 0.002785 |
| TFRC | 8.300503 | 0.002807 |
| CRAT | -5.6595 | 0.002831 |
| CD5 | -4.68472 | 0.002831 |
| ASCL1 | -3.20204 | 0.002831 |
| BRD3 | 6.241222 | 0.002831 |
| MAF | -5.86702 | 0.002851 |
| TNNI3 | 4.603825 | 0.002852 |
| NCK1 | 5.642491 | 0.002882 |
| EIF4B | -7.47713 | 0.002908 |
| CLTA | 7.22817 | 0.002908 |
| ENG | 5.582906 | 0.002928 |
| PNLIPRP1 | -3.50215 | 0.002997 |
| SLC35D1 | -5.97365 | 0.003018 |
| RFX5 | 6.39269 | 0.003022 |
| IGFBP3 | 8.434994 | 0.003022 |
| MAST1 | -4.66136 | 0.003027 |
| DNAH9 | -2.43416 | 0.003031 |
| PPP1R11 | -6.86924 | 0.003036 |
| HSD17B8 | -5.95729 | 0.003036 |
| PHKG2 | -3.84208 | 0.003036 |
| HINT1 | 8.29711 | 0.003036 |
| PRR3 | 4.348972 | 0.003059 |
| HOXB6 | 4.873866 | 0.003063 |
| SMAD4 | -4.51317 | 0.003083 |
| SYP | -3.78797 | 0.003086 |
| SPTB | -4.10026 | 0.003114 |
| SULT2B1 | 5.528511 | 0.003149 |
| ANP32B | 8.136589 | 0.003169 |
| PSMD1 | 7.303417 | 0.003243 |
| MMP7 | 9.460795 | 0.003277 |
| FPR2 | 4.204197 | 0.003279 |
| TROVE2 | 6.252699 | 0.003286 |
| ZNF250 | 3.685876 | 0.003286 |
| EEF1A1 | -10.486 | 0.003318 |
| ATP6V0D1 | -7.69076 | 0.003318 |
| FXR1 | 5.886592 | 0.003318 |
| RPL9 | -10.7313 | 0.003329 |
| ARHGEF12 | -4.15776 | 0.003354 |
| NPY6R | -4.11999 | 0.003358 |
| PLP1 | -4.28987 | 0.003381 |
| IQCB1 | 5.475838 | 0.003383 |
| PTPN7 | -4.70535 | 0.003386 |
| CHRNE | -3.58091 | 0.003387 |
| MXD1 | -7.61162 | 0.003399 |
| RNF113A | -5.96619 | 0.003407 |
| ARPC4 | 6.388468 | 0.003415 |
| HNRNPA1 | 6.308485 | 0.003426 |
| ASNA1 | 5.814226 | 0.003432 |
| STAT4 | -5.43528 | 0.003448 |
| MPP2 | -4.16023 | 0.003497 |
| IL17A | -3.51262 | 0.003527 |
| LIMK1 | 2.805096 | 0.003527 |
| VGLL4 | 6.66316 | 0.003528 |
| CNP | 5.499307 | 0.003538 |
| COL4A3 | -3.80636 | 0.003546 |
| PRSS8 | -8.03033 | 0.003549 |
| ARSA | -4.54933 | 0.003556 |
| TPM4 | 6.862194 | 0.003568 |
| MT3 | -4.51609 | 0.00358 |
| CLU | -7.68557 | 0.003587 |
| CSTF3 | 5.351439 | 0.003601 |
| EEF1D | 8.004252 | 0.003605 |
| NFIC | -6.94932 | 0.003624 |
| IL5RA | -3.14807 | 0.003624 |
| MYC | 8.47285 | 0.003624 |
| SERPINA1 | 9.213717 | 0.003624 |
| NUP153 | 6.12507 | 0.003632 |
| CENPB | 5.899714 | 0.003647 |
| JUN | -7.38184 | 0.003669 |
| LAMC1 | 7.54815 | 0.003669 |
| GOLGA1 | -4.46906 | 0.003674 |
| NDUFS1 | -5.91395 | 0.00369 |
| SLC4A2 | -6.63778 | 0.003704 |
| SUCLG1 | -7.27412 | 0.003709 |
| SLC39A9 | -4.79286 | 0.003709 |
| HSPA6 | 5.383314 | 0.003737 |
| GIP | -7.16749 | 0.003761 |
| SLC33A1 | -5.16332 | 0.003765 |
| KIAA0100 | 5.899961 | 0.003782 |
| VSNL1 | 7.710047 | 0.003786 |
| DIS3 | 4.579681 | 0.00383 |
| ENOSF1 | -6.26816 | 0.003836 |
| KCNAB1 | -3.54298 | 0.003857 |
| INA | -4.56089 | 0.003857 |
| LIG4 | -4.83353 | 0.00386 |
| PRIM1 | 5.793576 | 0.003863 |
| CDH17 | 9.572055 | 0.003864 |
| CXCL9 | 9.290454 | 0.00389 |
| C21orf2 | -4.12475 | 0.003906 |
| SLC30A3 | -2.07675 | 0.003906 |
| PKNOX1 | 3.22748 | 0.003923 |
| GK2 | -4.01542 | 0.00396 |
| UBE2N | 6.81567 | 0.003962 |
| RBM5 | -5.40517 | 0.003981 |
| TCTA | -5.80928 | 0.004011 |
| PFDN5 | -7.90234 | 0.00403 |
| MYH10 | 6.690243 | 0.004046 |
| CAPG | 7.606359 | 0.004059 |
| DHX8 | 4.185866 | 0.004074 |
| RRAS | 6.81743 | 0.004084 |
| SMPD1 | -4.07478 | 0.004086 |
| APOE | 8.272933 | 0.004086 |
| MMP10 | 5.536597 | 0.004092 |
| HDAC1 | 8.246055 | 0.00411 |
| LAGE3 | 6.597674 | 0.004117 |
| ECM1 | 7.841997 | 0.004135 |
| CDC7 | 5.589674 | 0.004152 |
| TEAD1 | 5.123109 | 0.004152 |
| ACAA1 | -6.25114 | 0.004195 |
| CDC37 | 6.434263 | 0.004198 |
| COX17 | -4.24984 | 0.004212 |
| RRAD | 3.712481 | 0.004256 |
| VIPR2 | -4.38869 | 0.004261 |
| CD79B | -4.46547 | 0.004296 |
| CSPG4 | 3.472889 | 0.004318 |
| PSME1 | 8.226534 | 0.004338 |
| AVPR1B | -4.44218 | 0.004358 |
| CHGB | -6.83325 | 0.004358 |
| WIPF2 | -5.94311 | 0.004358 |
| NOS1 | -2.82938 | 0.004376 |
| HCK | 6.223514 | 0.004376 |
| COX8A | -8.58314 | 0.00438 |
| FARSA | 5.58707 | 0.00438 |
| YARS | -6.14803 | 0.004383 |
| AKAP9 | -5.42171 | 0.004387 |
| NTF3 | -5.18104 | 0.004391 |
| NEUROD1 | -4.49997 | 0.004391 |
| HNRNPC | 6.900032 | 0.004391 |
| SLC5A3 | 5.187876 | 0.004403 |
| CTSO | -6.69154 | 0.004403 |
| TTF1 | 5.162559 | 0.004406 |
| NFYB | 4.784605 | 0.004418 |
| SSTR3 | -3.52212 | 0.004428 |
| CRIP1 | 8.93295 | 0.004431 |
| ITGA2B | -2.71059 | 0.004454 |
| SLC2A4 | -3.04299 | 0.004458 |
| SHB | 5.037468 | 0.004458 |
| NEK4 | 5.203538 | 0.004458 |
| BMPR1B | -5.01363 | 0.004462 |
| PGAM1 | 8.424683 | 0.004469 |
| BTC | -4.73549 | 0.004472 |
| CDKN2D | 2.985838 | 0.004472 |
| ABL2 | 4.367768 | 0.004553 |
| ATP5G3 | -8.41139 | 0.004561 |
| GFI1 | -4.28976 | 0.004561 |
| R3HDM1 | 5.849693 | 0.004561 |
| CDC25C | 3.508333 | 0.004581 |
| HGF | 3.350186 | 0.004601 |
| RALY | 6.787532 | 0.004605 |
| C4BPA | 5.353522 | 0.004612 |
| SECTM1 | -6.902 | 0.004614 |
| BRS3 | -3.14273 | 0.004614 |
| FIG4 | -5.57067 | 0.004616 |
| MAPK14 | 4.783198 | 0.004632 |
| SOX4 | 7.092674 | 0.004656 |
| MRPL3 | 8.145752 | 0.004656 |
| EVI2B | -7.59132 | 0.00466 |
| EPS15 | -6.60358 | 0.004662 |
| STXBP1 | 6.313755 | 0.004662 |
| P2RY10 | -4.85993 | 0.004668 |
| TAF1A | 3.916377 | 0.004668 |
| RBM38 | 6.047579 | 0.004668 |
| UBE2L3 | 6.579209 | 0.004668 |
| SRP9 | 8.337333 | 0.004668 |
| RABIF | 4.875329 | 0.004696 |
| TJP2 | -6.13562 | 0.00477 |
| PRLR | -4.24783 | 0.004788 |
| ICT1 | 5.885376 | 0.004788 |
| MPG | 5.70336 | 0.004813 |
| GNB2 | 7.612662 | 0.004841 |
| DAZAP2 | -7.35185 | 0.004845 |
| SLAMF1 | -4.00118 | 0.004853 |
| MAP3K12 | -5.15763 | 0.004866 |
| PDLIM4 | 4.680701 | 0.004868 |
| PSME3 | 6.17219 | 0.004907 |
| CHAF1A | 4.525115 | 0.004962 |
| MAOB | -5.38855 | 0.005034 |
| PDHX | -7.29701 | 0.005034 |
| MSN | 7.491583 | 0.005036 |
| LAPTM5 | 8.596214 | 0.005039 |
| TNFRSF4 | 4.000232 | 0.005065 |
| FOXO4 | -4.57538 | 0.005068 |
| CASP10 | -4.12423 | 0.005112 |
| JUP | 8.316086 | 0.005155 |
| EMG1 | 6.91341 | 0.005157 |
| RBM42 | 5.97222 | 0.005172 |
| TPM3 | 6.430508 | 0.005186 |
| COPS5 | 7.668151 | 0.005187 |
| RFTN1 | 6.707846 | 0.005195 |
| YAP1 | 7.072969 | 0.005209 |
| OLFM1 | -4.45151 | 0.005212 |
| CYP3A4 | -6.73055 | 0.005218 |
| NEUROD2 | -3.45528 | 0.005218 |
| CELSR2 | 5.07578 | 0.005218 |
| ABR | 5.454169 | 0.005289 |
| LAD1 | 7.034054 | 0.005292 |
| EPB41 | -4.67819 | 0.005335 |
| DMWD | -5.20041 | 0.005372 |
| CDH8 | -2.95105 | 0.005372 |
| PSMB6 | 7.52208 | 0.005373 |
| E2F1 | 4.284599 | 0.005401 |
| CETN1 | -4.37251 | 0.005421 |
| ITGAX | 4.357267 | 0.005499 |
| PRPS1 | 5.693443 | 0.005546 |
| NEO1 | -5.37256 | 0.005549 |
| OTUD4 | 5.140178 | 0.005593 |
| FMR1 | 6.66194 | 0.005689 |
| MMP2 | 7.356207 | 0.005716 |
| EPHA5 | -2.87555 | 0.005821 |
| SMARCD3 | -4.14612 | 0.005824 |
| MYOM1 | -3.93415 | 0.005824 |
| NEB | 5.373544 | 0.005824 |
| PSMB8 | 7.889978 | 0.005825 |
| KCNJ11 | -3.45423 | 0.00585 |
| MMRN1 | -5.3551 | 0.005855 |
| SLC39A14 | -7.15973 | 0.005882 |
| CNN3 | 7.347037 | 0.005908 |
| OGT | -5.87993 | 0.006002 |
| TAF4 | 5.858847 | 0.006016 |
| BAMBI | 7.534663 | 0.006026 |
| CXCL6 | 6.492761 | 0.006077 |
| GART | 4.230658 | 0.006122 |
| BPTF | 5.088621 | 0.006122 |
| RAD23A | 6.898679 | 0.006152 |
| DRAP1 | 6.005914 | 0.006172 |
| ITGB3 | -2.99274 | 0.006209 |
| TXN | 8.724632 | 0.006214 |
| OR2H1 | -2.56437 | 0.006229 |
| RYK | 5.768896 | 0.006321 |
| GLMN | 4.687084 | 0.006376 |
| CYP2A6 | -3.58665 | 0.006391 |
| SLC15A2 | -4.01176 | 0.006392 |
| MSH6 | 5.901668 | 0.006397 |
| KHDRBS1 | 6.554686 | 0.006399 |
| RND3 | 7.669627 | 0.006442 |
| FSHB | -4.20516 | 0.006448 |
| ZNHIT3 | 6.385559 | 0.006475 |
| EXT2 | 5.482358 | 0.006509 |
| TLN2 | -4.38648 | 0.006522 |
| MAPKAPK2 | -5.04784 | 0.006569 |
| TPD52 | -7.72248 | 0.006571 |
| RPS6KA3 | 6.388019 | 0.006605 |
| CTSC | 7.894104 | 0.006623 |
| BDKRB2 | 5.553179 | 0.006634 |
| ZNF140 | -5.46558 | 0.006657 |
| MBTPS1 | -5.29903 | 0.00671 |
| GRIN2B | -2.60001 | 0.00671 |
| CD86 | 5.427502 | 0.00671 |
| COPS6 | 7.144217 | 0.006713 |
| SOD1 | 9.227518 | 0.006713 |
| TFAP2B | -2.64117 | 0.006717 |
| PMAIP1 | 6.787581 | 0.006736 |
| CD3E | -5.71351 | 0.006766 |
| GABRA1 | -2.50694 | 0.006782 |
| HOXB1 | -3.86874 | 0.006786 |
| F2RL2 | 6.296086 | 0.006804 |
| IFRD2 | -6.79842 | 0.006851 |
| PAFAH1B2 | 5.593499 | 0.006871 |
| VRK2 | 6.374367 | 0.006876 |
| SERPINB2 | 7.387379 | 0.006895 |
| GH1 | -3.3982 | 0.006979 |
| NUP93 | 4.660466 | 0.006979 |
| PF4 | 4.839448 | 0.006999 |
| CTNNA1 | -7.40954 | 0.00702 |
| SH3GL3 | -3.31957 | 0.007036 |
| PPIF | -7.09216 | 0.00704 |
| VPS45 | -4.7671 | 0.00704 |
| PREP | 6.089825 | 0.00708 |
| MYCN | -4.03101 | 0.007083 |
| EGR2 | 6.072526 | 0.007151 |
| F2RL1 | -8.17557 | 0.007234 |
| SREBF2 | -4.7278 | 0.007255 |
| OSM | 5.10781 | 0.007292 |
| KIAA0196 | 5.942308 | 0.007292 |
| SEPW1 | 8.213205 | 0.007292 |
| GTF2H4 | 4.560742 | 0.007305 |
| SLC18A2 | -4.19334 | 0.007314 |
| ACRV1 | -3.64576 | 0.007322 |
| HMGCS2 | -7.6036 | 0.007323 |
| RAC1 | 7.680454 | 0.007461 |
| PHLDA2 | 7.670879 | 0.007465 |
| MFGE8 | 7.3316 | 0.007471 |
| SRD5A1 | 5.789299 | 0.007578 |
| TNP1 | -3.75514 | 0.007596 |
| CCNE1 | 6.665266 | 0.007614 |
| SLC7A5 | 7.373208 | 0.007635 |
| ATM | -4.48801 | 0.007635 |
| COL13A1 | -3.84719 | 0.007635 |
| EIF2S2 | 7.500354 | 0.007667 |
| CDK7 | 6.517624 | 0.007787 |
| HRH1 | 5.072976 | 0.007788 |
| CHRM5 | -4.73153 | 0.007847 |
| NNAT | -4.92731 | 0.007869 |
| MAS1 | -4.72525 | 0.007944 |
| NDUFV3 | -6.62961 | 0.00795 |
| USP9Y | -4.90748 | 0.00795 |
| MAP3K8 | 5.473035 | 0.007958 |
| RPS23 | -9.866 | 0.007964 |
| RARB | -3.1352 | 0.008011 |
| GALNS | 4.274087 | 0.00825 |
| TSN | 6.661966 | 0.008293 |
| SOX11 | 3.698381 | 0.008328 |
| SF1 | 5.385227 | 0.008361 |
| TAF1B | 4.129736 | 0.008377 |
| C3AR1 | 6.352379 | 0.008377 |
| IL6 | 6.831771 | 0.008446 |
| SERPINB9 | 6.500374 | 0.008457 |
| ADRBK1 | -4.42843 | 0.008485 |
| AHCY | 7.878201 | 0.008485 |
| NMU | 7.866293 | 0.00853 |
| RPS4Y1 | -9.929 | 0.008544 |
| PITX1 | 4.240232 | 0.008567 |
| EIF2B5 | 4.661718 | 0.008573 |
| CLPS | -5.06608 | 0.008598 |
| TRAP1 | 6.26858 | 0.008598 |
| UCHL3 | 7.251536 | 0.008598 |
| SP2 | -4.76296 | 0.008605 |
| NAGA | 5.29114 | 0.008605 |
| EMP1 | 7.177175 | 0.008612 |
| FRMPD4 | -2.94 | 0.008756 |
| CIB1 | 8.007981 | 0.008793 |
| THBS4 | 8.06218 | 0.008795 |
| FAS | 6.246802 | 0.008809 |
| CTDSP2 | -6.35234 | 0.008818 |
| ZMYND8 | -5.46163 | 0.008818 |
| DUSP6 | 7.590674 | 0.008818 |
| PTK7 | 4.500175 | 0.008897 |
| MAP2K1 | 6.110532 | 0.008897 |
| ARHGEF6 | -6.23011 | 0.008897 |
| MBP | -4.18681 | 0.008897 |
| CYLC2 | -3.18304 | 0.008897 |
| USP10 | 5.771253 | 0.008897 |
| RPL18 | 8.794869 | 0.008897 |
| FEV | -4.29734 | 0.008911 |
| G3BP1 | 5.247958 | 0.008911 |
| CASP8 | 5.218679 | 0.008979 |
| MX2 | -6.53517 | 0.008986 |
| UBE2B | -5.67469 | 0.008986 |
| TNFAIP3 | 7.853458 | 0.008986 |
| P4HA1 | 5.840517 | 0.009006 |
| DEFA6 | -9.63815 | 0.009019 |
| PROS1 | 6.847034 | 0.009099 |
| EPHX1 | -7.31208 | 0.00914 |
| PRTN3 | -3.12689 | 0.00914 |
| HSPA9 | 6.611293 | 0.00914 |
| PSMA5 | 6.68735 | 0.00914 |
| EPRS | 6.78708 | 0.00914 |
| TIE1 | 4.053216 | 0.009156 |
| CNGA1 | -4.59858 | 0.009223 |
| GP9 | -4.02429 | 0.009223 |
| PPARG | -6.81245 | 0.009246 |
| CXCL11 | 7.601381 | 0.009246 |
| PMP22 | 6.815946 | 0.009311 |
| KIAA0020 | 5.270402 | 0.009317 |
| RPL11 | -9.2441 | 0.009442 |
| SLC35B1 | -6.73806 | 0.009442 |
| ATP6V1B1 | -3.3183 | 0.009442 |
| GRB14 | 5.085618 | 0.009442 |
| ADAM10 | 6.644809 | 0.009453 |
| NEFM | -2.57371 | 0.009456 |
| GYPA | -2.0413 | 0.009467 |
| LAMB1 | 7.381699 | 0.009475 |
| KITLG | -6.22313 | 0.009503 |
| ZNF76 | -3.61342 | 0.009542 |
| TAGLN | 8.827569 | 0.009542 |
| CXCL3 | 7.821247 | 0.00956 |
| C8B | -4.64349 | 0.00961 |
| CKAP4 | -7.16541 | 0.009612 |
| SLC4A1 | -3.56347 | 0.009613 |
| EIF5A | 7.869578 | 0.009661 |
| AREG | 6.602541 | 0.009661 |
| CAMK1 | 4.279036 | 0.009683 |
| TCEA1 | 6.59083 | 0.009726 |
| ETV3 | -5.23399 | 0.009745 |
| DSC2 | -7.48186 | 0.009822 |
| RRM1 | 7.124382 | 0.009835 |
| HTR7 | -3.17575 | 0.009881 |
| RPS25 | -9.43885 | 0.009939 |
| RUNX2 | -4.6568 | 0.009949 |
| NXF1 | -5.71458 | 0.009967 |
| ANXA3 | -8.21111 | 0.01 |
| CDK9 | 3.869656 | 0.010028 |
| ECE1 | 4.40353 | 0.010094 |
| PPP2R2A | 5.783622 | 0.010094 |
| MTIF2 | 6.513938 | 0.010099 |
| HAS2 | 6.068779 | 0.010117 |
| RPL39 | -10.9189 | 0.010247 |
| CDH4 | -3.22194 | 0.010249 |
| FAM50A | 6.227091 | 0.010291 |
| CD1D | -5.066 | 0.010322 |
| ALPI | -4.30441 | 0.010522 |
| CD93 | 5.962346 | 0.010522 |
| RPS5 | 9.018018 | 0.010545 |
| SCRN1 | 6.888562 | 0.010603 |
| GNAI1 | -5.90082 | 0.010614 |
| ARSE | 7.079534 | 0.010639 |
| LRP1 | 4.388706 | 0.010661 |
| SLC1A4 | -5.07647 | 0.010682 |
| STARD9 | -3.71764 | 0.010683 |
| SNX17 | 6.299507 | 0.010683 |
| ANXA13 | 7.333691 | 0.010693 |
| ALAS2 | -2.95096 | 0.010717 |
| SF3B2 | 5.28101 | 0.010726 |
| BLMH | 5.393125 | 0.01075 |
| HCG9 | -4.12736 | 0.010767 |
| ZNF174 | 3.150389 | 0.010767 |
| SUZ12 | 5.850655 | 0.010769 |
| IDH2 | -7.96493 | 0.010841 |
| PTGER4 | -7.11361 | 0.010841 |
| YWHAZ | 8.247465 | 0.010863 |
| PNLIP | -6.38105 | 0.010884 |
| PTGER2 | -6.74382 | 0.010893 |
| VPS72 | 5.970406 | 0.0109 |
| SMAD3 | -4.35277 | 0.010902 |
| CD19 | -6.23795 | 0.010956 |
| TAF5 | 4.655737 | 0.01102 |
| WWP1 | -6.5937 | 0.011088 |
| PLXNB1 | 4.036409 | 0.011095 |
| CETN2 | 6.849706 | 0.011104 |
| GPLD1 | -2.79143 | 0.011175 |
| SRPK1 | 6.584199 | 0.011195 |
| GBP1 | 8.192109 | 0.011229 |
| GRM4 | -4.0458 | 0.011236 |
| MAMLD1 | -4.24995 | 0.011252 |
| PPIL2 | 3.523549 | 0.011252 |
| CST6 | 5.794009 | 0.011252 |
| IFIT1 | 7.395004 | 0.011304 |
| NQO2 | 6.067228 | 0.011316 |
| MPI | -4.44003 | 0.011355 |
| ERCC3 | 4.626342 | 0.011355 |
| PML | 3.801989 | 0.01136 |
| ZNF141 | -3.46735 | 0.011377 |
| ETS2 | 7.235512 | 0.011419 |
| FXYD3 | -8.6017 | 0.011448 |
| RPGR | 4.340637 | 0.011476 |
| SHROOM2 | -4.74555 | 0.011588 |
| SERPINF1 | 8.410379 | 0.011588 |
| MAP4K1 | -4.85517 | 0.011604 |
| DIO2 | 5.95934 | 0.011657 |
| IDI1 | -6.69421 | 0.011841 |
| MGMT | -5.44694 | 0.011841 |
| RPS17 | -9.63139 | 0.011861 |
| GLO1 | 8.15382 | 0.011861 |
| TP53I11 | 4.277049 | 0.011863 |
| SLC12A3 | -2.62571 | 0.011915 |
| MYL2 | -2.87065 | 0.011917 |
| MLLT3 | -4.67573 | 0.011935 |
| CRYGD | -3.79939 | 0.012021 |
| FGFR1 | 4.179878 | 0.012031 |
| EFNA1 | 7.880208 | 0.012047 |
| IL13RA1 | 6.170242 | 0.012069 |
| RLF | 4.640866 | 0.012163 |
| FGF9 | -4.33355 | 0.012258 |
| KIF5B | 7.624981 | 0.012258 |
| COMT | 6.479042 | 0.012491 |
| GTF2A2 | 6.604353 | 0.012519 |
| FGF4 | -3.85811 | 0.012596 |
| CCT8 | 7.236946 | 0.012631 |
| GPX2 | -8.89278 | 0.012732 |
| ASGR2 | 5.486268 | 0.012804 |
| TAL1 | -3.37867 | 0.012853 |
| SMAD6 | -4.08951 | 0.01288 |
| CCL7 | 3.724122 | 0.012889 |
| HBZ | -3.27293 | 0.012892 |
| CEACAM5 | 9.813875 | 0.013002 |
| TBX2 | -3.99453 | 0.013021 |
| GNB3 | -3.34202 | 0.013036 |
| PLP2 | 7.884492 | 0.013094 |
| MARK3 | -4.59201 | 0.013113 |
| PLAC4 | -3.76771 | 0.013113 |
| CACNA1S | -4.27206 | 0.013117 |
| EWSR1 | 5.284943 | 0.013266 |
| SLC6A6 | 4.795373 | 0.013271 |
| MGAT3 | -3.78076 | 0.013312 |
| IL13 | -4.34142 | 0.013312 |
| RCOR1 | 5.818539 | 0.013356 |
| KLK6 | 7.207145 | 0.013356 |
| IL18 | 6.347084 | 0.013383 |
| VCAM1 | 7.244392 | 0.01339 |
| TMEM97 | -7.94449 | 0.013455 |
| HMGCS1 | -6.96507 | 0.013505 |
| MDM2 | 4.344157 | 0.013552 |
| TYRO3 | -4.3759 | 0.013592 |
| SYCP1 | -2.86991 | 0.013765 |
| PITPNB | 6.791725 | 0.013774 |
| SLC20A1 | 7.180272 | 0.013787 |
| IFI44L | 7.08469 | 0.01383 |
| SLBP | 7.061118 | 0.013859 |
| HARS2 | -5.07895 | 0.014047 |
| SPTLC2 | -5.02001 | 0.014084 |
| S100A2 | 9.023187 | 0.014168 |
| CUL2 | 4.708683 | 0.01419 |
| LILRB1 | 5.257948 | 0.014262 |
| POLD3 | 5.134445 | 0.014294 |
| TNC | 6.738223 | 0.014344 |
| TCF19 | 4.885734 | 0.014443 |
| ZNF184 | 4.885477 | 0.014448 |
| IGFBP5 | -6.79301 | 0.014536 |
| SLC23A2 | -3.7542 | 0.014553 |
| ATP6AP1 | 7.034905 | 0.014617 |
| PDHB | -7.1283 | 0.014764 |
| REN | -4.22762 | 0.014764 |
| AR | -3.89721 | 0.014764 |
| YWHAE | 7.311846 | 0.014802 |
| GDNF | -3.10363 | 0.014845 |
| FOSL1 | 6.235846 | 0.014901 |
| GABRA5 | -3.34913 | 0.014949 |
| DSG3 | 7.446175 | 0.014995 |
| CHRNG | -4.06955 | 0.014999 |
| HIST1H2BE | 6.87826 | 0.015061 |
| TOPBP1 | 6.005866 | 0.015117 |
| CDK8 | 5.425592 | 0.015275 |
| CP | 6.710164 | 0.015307 |
| ELN | 5.100798 | 0.015492 |
| U2AF2 | 5.405997 | 0.015523 |
| FOXF1 | -6.59711 | 0.015633 |
| LAMP1 | 7.53531 | 0.015706 |
| PCCB | -5.32581 | 0.015762 |
| IL1RL1 | -3.9128 | 0.015791 |
| CSNK2A1 | 6.156703 | 0.015809 |
| HIST1H2BH | 6.480283 | 0.015809 |
| DLD | -7.27172 | 0.01585 |
| LCP2 | 5.215688 | 0.015885 |
| UPF1 | 4.576723 | 0.015956 |
| VIPR1 | -5.6303 | 0.016032 |
| CTF1 | -4.06597 | 0.016045 |
| TRIM14 | 5.289348 | 0.016076 |
| SLC31A2 | -5.78202 | 0.016107 |
| SLC7A1 | 5.66073 | 0.016324 |
| RHOC | 6.914512 | 0.016368 |
| ICAM3 | -6.21506 | 0.01639 |
| VIL1 | 7.279749 | 0.016397 |
| MPZ | -3.88454 | 0.016494 |
| CRYAA | -2.13491 | 0.016494 |
| TGFBR2 | -6.72244 | 0.016509 |
| PDGFA | 5.392886 | 0.016519 |
| KRT33B | 2.457347 | 0.016529 |
| THRB | -4.64915 | 0.016533 |
| PTPRR | -4.97181 | 0.016598 |
| PYY | -3.06767 | 0.016672 |
| C1orf61 | -3.30682 | 0.016791 |
| LTA | -2.53669 | 0.016825 |
| DBP | -4.18225 | 0.016831 |
| GNS | 6.648467 | 0.016879 |
| KCTD2 | 4.265461 | 0.017087 |
| DARS | 6.798943 | 0.017087 |
| BLM | 5.498081 | 0.017111 |
| EIF3I | 7.083517 | 0.017111 |
| JUND | -6.7935 | 0.017139 |
| HOXA4 | 4.202638 | 0.017139 |
| RPUSD2 | 4.656215 | 0.017139 |
| ACTN2 | -2.48396 | 0.017167 |
| HMGN2 | 8.258354 | 0.017167 |
| ARHGAP25 | -4.90477 | 0.017172 |
| GBP2 | 6.549233 | 0.017172 |
| CAPNS1 | 7.976363 | 0.017203 |
| RARS | 7.182796 | 0.017225 |
| SOX5 | -3.06205 | 0.017306 |
| TXN2 | -5.89851 | 0.017344 |
| FLI1 | -4.7481 | 0.017401 |
| WNT10B | -3.59307 | 0.017416 |
| TWF1 | 5.980273 | 0.017416 |
| TROAP | 5.274367 | 0.017423 |
| PIP5K1B | -6.56488 | 0.017456 |
| STXBP2 | -4.7549 | 0.017456 |
| SMCP | -1.49943 | 0.017458 |
| CRKL | 5.435766 | 0.017468 |
| PRODH2 | -4.10777 | 0.017483 |
| HTR2A | -3.24678 | 0.01753 |
| NEDD4 | 4.674791 | 0.01767 |
| CDX2 | 5.051835 | 0.017773 |
| SNAPC1 | 5.301759 | 0.017773 |
| TNFAIP1 | 5.163038 | 0.017841 |
| PRKD1 | -3.61756 | 0.017855 |
| MSX1 | 4.560025 | 0.017855 |
| B4GALNT1 | -3.00502 | 0.01788 |
| IVL | -4.14968 | 0.017994 |
| KRT2 | -4.11002 | 0.018227 |
| ELAVL3 | -1.98691 | 0.018237 |
| CTCF | 5.913869 | 0.01843 |
| ARL1 | -6.15738 | 0.018511 |
| NMB | 5.899822 | 0.018566 |
| RNPEP | 7.400979 | 0.018584 |
| SP3 | 5.224196 | 0.018623 |
| CD47 | 7.030309 | 0.018651 |
| USP7 | 5.768576 | 0.018719 |
| RGS6 | -1.6382 | 0.018754 |
| RPLP0 | -9.36797 | 0.018778 |
| KCNMB1 | -5.37829 | 0.01881 |
| MAPK8 | 3.817978 | 0.018864 |
| SMARCA2 | -5.0028 | 0.018891 |
| MAPK7 | -3.75861 | 0.018891 |
| SLC6A4 | -3.05296 | 0.018903 |
| OR1D2 | -4.23821 | 0.018904 |
| AGTR2 | -2.20735 | 0.018906 |
| RARRES2 | 7.490542 | 0.018983 |
| BIRC3 | -7.47851 | 0.019004 |
| RENBP | 3.91853 | 0.01903 |
| LAMP2 | -6.99859 | 0.019043 |
| LIPA | 8.255643 | 0.019043 |
| GRIA2 | -1.8485 | 0.0193 |
| ADCY7 | 4.953278 | 0.0193 |
| CHRNB3 | -2.99985 | 0.019345 |
| YME1L1 | 5.657719 | 0.019362 |
| NCOR2 | 5.350203 | 0.019464 |
| SEMG1 | 5.725916 | 0.019464 |
| MADD | -4.60268 | 0.019554 |
| CLCN3 | -6.36248 | 0.019597 |
| POLB | 5.740085 | 0.019597 |
| UBE2G1 | -5.88865 | 0.019871 |
| KIR2DL4 | -2.99088 | 0.019871 |
| GNA15 | 5.126011 | 0.019871 |
| DDB2 | 6.07059 | 0.019871 |
| CGRRF1 | -4.60467 | 0.019923 |
| SKIL | 5.579684 | 0.019953 |
| PPP5C | 4.16272 | 0.020044 |
| GLG1 | 6.243971 | 0.020044 |
| FABP2 | -4.87883 | 0.020134 |
| PKP1 | -2.81688 | 0.020299 |
| CACNA1B | -2.50125 | 0.020504 |
| CTH | -5.36014 | 0.020534 |
| GAL3ST1 | -4.42968 | 0.020534 |
| ALKBH1 | -4.29458 | 0.020534 |
| GRIN1 | -3.16126 | 0.020534 |
| IL4R | 5.831159 | 0.020534 |
| SFN | 9.381509 | 0.020534 |
| UBE2V2 | 5.32821 | 0.020658 |
| CLCN4 | 3.19718 | 0.020805 |
| PENK | 3.638143 | 0.020805 |
| CD48 | -6.6043 | 0.020829 |
| NOS3 | 3.24337 | 0.020924 |
| GFPT1 | -7.27489 | 0.021044 |
| PPP3CC | -3.79568 | 0.02107 |
| SRP19 | -6.93598 | 0.021157 |
| NOVA1 | -3.0178 | 0.021185 |
| COL11A2 | -3.92227 | 0.021188 |
| JARID2 | -4.78377 | 0.021286 |
| CCDC130 | -4.5595 | 0.021374 |
| HOXB2 | 6.262853 | 0.021435 |
| AGT | 7.747128 | 0.021453 |
| FABP6 | 4.290798 | 0.021714 |
| OCLN | -5.77482 | 0.021769 |
| IFT88 | 5.324159 | 0.021769 |
| SCN7A | -3.88164 | 0.021834 |
| TACR1 | -1.74645 | 0.021834 |
| UBAP2L | 5.323063 | 0.021887 |
| STX5 | -4.48811 | 0.021951 |
| IFI27 | -9.52577 | 0.022155 |
| OAT | -9.17348 | 0.022198 |
| TEP1 | -5.44341 | 0.022294 |
| KCNJ10 | -3.14574 | 0.022314 |
| SLC9A3 | -2.32826 | 0.022321 |
| SRD5A2 | -2.95951 | 0.022337 |
| MTM1 | -4.29988 | 0.02234 |
| TLR4 | 4.066161 | 0.02234 |
| CA7 | -3.87228 | 0.022405 |
| ZNF212 | -4.59389 | 0.022439 |
| CCR9 | -3.08127 | 0.022555 |
| ASNS | -6.40706 | 0.02265 |
| KCNAB2 | 3.88875 | 0.022663 |
| SMOX | -3.9209 | 0.022734 |
| LILRA2 | 3.136741 | 0.022734 |
| AP3M2 | 5.069577 | 0.022737 |
| MYL9 | 7.961907 | 0.022751 |
| FBXO21 | -5.8031 | 0.022857 |
| TFPI | 6.684228 | 0.022994 |
| VBP1 | 6.744354 | 0.023186 |
| NPY5R | -3.41518 | 0.023234 |
| PRAME | 5.613686 | 0.023309 |
| MVD | -3.94512 | 0.023395 |
| AP3D1 | -5.4809 | 0.023447 |
| RELN | -4.2025 | 0.023534 |
| MC5R | -3.01192 | 0.023534 |
| MAX | 5.410734 | 0.023588 |
| GRIN2A | -2.9648 | 0.023626 |
| CYFIP1 | 6.841661 | 0.02396 |
| CEACAM7 | 8.043955 | 0.023965 |
| BRD8 | 4.549437 | 0.024026 |
| KCNJ5 | -3.48337 | 0.024033 |
| MAML1 | 5.50997 | 0.024095 |
| SMTN | 5.95508 | 0.024095 |
| EIF1 | -7.72544 | 0.02411 |
| PSMD7 | 6.794016 | 0.024125 |
| CALR | -7.44328 | 0.024143 |
| APBB2 | 4.041231 | 0.024188 |
| GHRH | -3.96002 | 0.024453 |
| KLK7 | 5.789039 | 0.024453 |
| GNB1 | 6.842682 | 0.024453 |
| RGS3 | 4.540644 | 0.024795 |
| ENO2 | 5.800522 | 0.024833 |
| PRSS3 | 8.437255 | 0.024884 |
| PMCH | 2.786274 | 0.025073 |
| SKIV2L2 | -5.1117 | 0.025156 |
| ALDOA | 8.399966 | 0.025196 |
| KCNK1 | -7.00289 | 0.025228 |
| TXLNA | 4.260593 | 0.025387 |
| ALPL | -3.57237 | 0.025521 |
| BIRC2 | 6.402713 | 0.02557 |
| RXRG | -3.81553 | 0.025665 |
| SLC12A2 | -8.14941 | 0.02598 |
| MYF5 | -3.0569 | 0.026208 |
| SEC23A | 6.579054 | 0.026255 |
| C8A | -2.91899 | 0.026315 |
| PLEK | 6.337108 | 0.026315 |
| PHB2 | 8.343914 | 0.026315 |
| GSTO1 | 7.608402 | 0.026339 |
| CEACAM1 | 6.794849 | 0.026359 |
| HNRNPL | 7.576289 | 0.026359 |
| FGF7 | 2.904599 | 0.026428 |
| FDX1 | -5.89452 | 0.026565 |
| HIP1 | 3.738891 | 0.026594 |
| OAS2 | 5.469025 | 0.026661 |
| ART1 | -3.17628 | 0.026809 |
| WT1 | 4.079866 | 0.026832 |
| HOXB5 | 4.326418 | 0.026832 |
| GGT5 | 4.025862 | 0.027054 |
| POLA1 | 4.664006 | 0.027177 |
| CLN3 | -5.37151 | 0.027248 |
| ST3GAL1 | -5.13351 | 0.027273 |
| PTPRM | -5.85306 | 0.027348 |
| NCAPD3 | 5.698911 | 0.027433 |
| CMKLR1 | 3.810944 | 0.02748 |
| GNAI3 | 5.888957 | 0.0275 |
| NKX3-1 | -3.46603 | 0.027569 |
| RPS21 | 9.298499 | 0.027594 |
| MAN2B1 | 6.490636 | 0.027693 |
| ABAT | -6.11302 | 0.027737 |
| H2BFXP | 2.460178 | 0.027839 |
| CAPRIN1 | 6.428629 | 0.027862 |
| CCR4 | -4.04955 | 0.027972 |
| TIMM44 | 4.290476 | 0.028282 |
| EN2 | 3.785887 | 0.028918 |
| GUCY2D | -3.23335 | 0.028944 |
| SERPINA3 | 9.265724 | 0.029062 |
| ANKRD46 | -5.77946 | 0.02913 |
| AMELX | -3.61792 | 0.029157 |
| S100A7 | 6.628242 | 0.029208 |
| BCL2 | -4.01112 | 0.029363 |
| TSG101 | -6.59353 | 0.029462 |
| CNTN2 | -2.22244 | 0.029462 |
| GNAL | 3.65673 | 0.029462 |
| EPHA3 | 4.766154 | 0.029462 |
| KNTC1 | 4.965461 | 0.029462 |
| GJA1 | 8.519724 | 0.029462 |
| DPF1 | -3.45768 | 0.029502 |
| TM9SF1 | -4.99939 | 0.029511 |
| TMOD1 | -3.99068 | 0.029623 |
| RBBP8 | 6.207738 | 0.029623 |
| FCN2 | -2.60457 | 0.029773 |
| PPP1R1A | -4.18655 | 0.029847 |
| INPP4B | 4.373231 | 0.030043 |
| RASA1 | -5.59651 | 0.030089 |
| PMP2 | -2.45765 | 0.030175 |
| IGF2R | 6.14165 | 0.030428 |
| CHRM1 | -3.25384 | 0.030528 |
| CPSF6 | 5.19547 | 0.030656 |
| SLC30A1 | 5.440676 | 0.030656 |
| PRKCG | -3.21361 | 0.030657 |
| PRNP | 7.396914 | 0.030687 |
| CSF3 | 3.575934 | 0.031073 |
| ECH1 | -7.05721 | 0.031216 |
| LTBR | 4.490761 | 0.031406 |
| ARFIP2 | -6.18659 | 0.031482 |
| DPP6 | -2.97681 | 0.031503 |
| STX16 | 5.769919 | 0.031938 |
| TOB2 | 5.756817 | 0.031959 |
| GH2 | -3.33368 | 0.032026 |
| KPNA1 | 4.429866 | 0.032148 |
| MGST1 | -7.31704 | 0.032152 |
| AXIN1 | 4.590775 | 0.032155 |
| PYGM | -3.56397 | 0.032629 |
| MPHOSPH6 | 5.351845 | 0.032629 |
| CTSD | 7.111272 | 0.032676 |
| SDHB | -5.91517 | 0.032719 |
| POLD1 | 5.176991 | 0.032719 |
| SLC8A1 | 2.809717 | 0.032776 |
| MPHOSPH9 | 3.881874 | 0.032776 |
| TAT | -2.74866 | 0.03285 |
| LRRC41 | 3.950497 | 0.03285 |
| CUL4A | 5.378558 | 0.032917 |
| INPP1 | 7.043077 | 0.033147 |
| TK1 | 6.131346 | 0.033314 |
| RPS27A | -8.50467 | 0.033394 |
| MMP13 | 3.331426 | 0.033406 |
| TDG | 6.61601 | 0.033534 |
| HBB | -9.47224 | 0.033566 |
| CHN2 | -4.58264 | 0.033569 |
| TGFA | -4.58533 | 0.033722 |
| MAGEA11 | 4.291738 | 0.033722 |
| SGSH | -4.65639 | 0.033806 |
| STARD8 | -4.59207 | 0.033873 |
| EEA1 | 4.242717 | 0.033948 |
| MPV17 | 5.071051 | 0.034004 |
| DECR1 | -7.28336 | 0.03402 |
| NFATC3 | 3.838642 | 0.03418 |
| DUT | 6.906038 | 0.034326 |
| FHL2 | -7.80459 | 0.034346 |
| APBA2 | 3.841493 | 0.034346 |
| FPR1 | 5.415056 | 0.03446 |
| NT5C2 | 6.949574 | 0.03446 |
| PARG | 4.718388 | 0.034659 |
| BLVRA | 5.61194 | 0.034901 |
| SRP54 | -5.57351 | 0.034986 |
| LDHC | 3.517248 | 0.034986 |
| MSX2 | 5.071355 | 0.034997 |
| POLR2H | 6.859101 | 0.035026 |
| TNNC1 | 4.531188 | 0.035097 |
| DUSP2 | 5.120485 | 0.035097 |
| ACR | -3.03394 | 0.035176 |
| PCMT1 | 6.124797 | 0.035176 |
| GRM5 | -2.59168 | 0.035424 |
| IRF7 | 6.155328 | 0.035456 |
| TRAF6 | -3.86786 | 0.035469 |
| ZNF132 | -3.08138 | 0.035469 |
| XRCC1 | 4.174721 | 0.035519 |
| GFAP | -2.00958 | 0.035644 |
| SEMA3F | -4.88541 | 0.035677 |
| TSHR | -2.07346 | 0.036083 |
| EZH1 | -3.78019 | 0.036253 |
| PSEN2 | 3.446031 | 0.036272 |
| NRIP1 | 6.771513 | 0.036291 |
| ABCA4 | -2.2598 | 0.036306 |
| CRYBA4 | -1.85766 | 0.036306 |
| SLC6A14 | 7.697648 | 0.036306 |
| CRYBB1 | -1.67896 | 0.03632 |
| MCL1 | 6.848048 | 0.03657 |
| TNFRSF14 | -5.27206 | 0.036601 |
| CLNS1A | 5.99245 | 0.036638 |
| LARS2 | 5.1556 | 0.036686 |
| POLR3C | 5.107404 | 0.037055 |
| ZNF74 | 2.531078 | 0.037416 |
| AQP6 | -2.9938 | 0.037421 |
| GRM8 | -2.52352 | 0.037461 |
| TUB | -2.90487 | 0.037503 |
| CEBPZ | 6.066851 | 0.037503 |
| WARS | 8.55546 | 0.037503 |
| SLC1A5 | 6.608918 | 0.03769 |
| MATN1 | -2.94685 | 0.037721 |
| DAP | -6.59684 | 0.037736 |
| BET1 | -5.954 | 0.03787 |
| RDX | -5.48431 | 0.038042 |
| FABP4 | -7.75521 | 0.038052 |
| MEF2C | -5.84027 | 0.038265 |
| JUNB | 7.729858 | 0.038341 |
| RORA | -3.62569 | 0.038353 |
| FOLR2 | -4.56963 | 0.038419 |
| SLC6A1 | 3.088412 | 0.038585 |
| ELF4 | -5.40919 | 0.038763 |
| SSTR4 | -3.19867 | 0.03884 |
| DLST | -5.93654 | 0.038884 |
| ABO | -3.35003 | 0.039019 |
| CREBBP | 4.606379 | 0.039099 |
| ABCD2 | -2.8654 | 0.039108 |
| NRG1 | -2.55374 | 0.039255 |
| RPS3 | 9.397797 | 0.039255 |
| EPHX2 | -5.97586 | 0.039662 |
| PALM | -4.66454 | 0.039662 |
| DCTN1 | -5.30195 | 0.040148 |
| NPEPPS | 5.117625 | 0.040191 |
| KLK2 | -2.68037 | 0.040326 |
| EGR4 | -3.0502 | 0.040438 |
| MLLT11 | 7.020639 | 0.040438 |
| S100A1 | -4.78549 | 0.040519 |
| MAPK9 | -4.66014 | 0.040596 |
| PTPRU | 4.539377 | 0.040799 |
| GMFB | 6.131208 | 0.040799 |
| ACP5 | 6.94866 | 0.040843 |
| ACAA2 | 5.868517 | 0.04085 |
| GRB10 | 5.128708 | 0.040854 |
| GAST | -9.81821 | 0.040924 |
| HRC | -2.76737 | 0.040954 |
| FMO1 | 5.082931 | 0.040954 |
| TBC1D5 | 5.054013 | 0.041243 |
| SNRPA1 | 6.789229 | 0.041243 |
| C19orf57 | 3.600014 | 0.041375 |
| EPHB4 | 5.653937 | 0.041375 |
| GNA11 | -6.82497 | 0.041411 |
| SEC24C | 5.802149 | 0.041707 |
| MCHR1 | -2.29035 | 0.041817 |
| SAP18 | 6.803834 | 0.041817 |
| PSMA2 | 7.124897 | 0.041869 |
| PSMD6 | 5.831803 | 0.041895 |
| PGR | -3.05091 | 0.042045 |
| GLP1R | -3.28852 | 0.042135 |
| PFKFB3 | 6.743396 | 0.042247 |
| PPARA | -3.43386 | 0.042248 |
| POU3F4 | -3.45976 | 0.042363 |
| PTPRC | 6.534716 | 0.042529 |
| GALNT3 | -7.51057 | 0.042557 |
| GRK4 | -2.0829 | 0.042977 |
| P2RX3 | -3.34017 | 0.042979 |
| SIX1 | 3.584578 | 0.043172 |
| FGR | 5.235879 | 0.04319 |
| TNNT2 | -2.81125 | 0.043254 |
| ARL4D | -3.36088 | 0.043321 |
| TBCE | 5.537807 | 0.043745 |
| SRRM2 | -6.23054 | 0.04389 |
| DLG4 | -2.78897 | 0.044011 |
| MSH3 | -4.72735 | 0.044094 |
| OXTR | 4.042746 | 0.044094 |
| HOXA5 | 5.255402 | 0.044146 |
| EHMT2 | 3.927639 | 0.04418 |
| COX11 | 5.380408 | 0.044605 |
| INPP5E | 5.323611 | 0.044905 |
| ITGA3 | 5.596448 | 0.045055 |
| ERCC5 | -5.10285 | 0.045156 |
| TBR1 | -2.87567 | 0.045182 |
| TRDN | -1.46258 | 0.045182 |
| ZNF3 | 3.510974 | 0.045182 |
| VAV2 | 4.284065 | 0.045182 |
| ADRB3 | -2.36509 | 0.045574 |
| UBL4A | 4.698385 | 0.045698 |
| TMED10 | -7.19847 | 0.045959 |
| TCP10 | -3.4179 | 0.046209 |
| ITGB2 | 6.378985 | 0.046245 |
| NAPA | -5.81892 | 0.046254 |
| SSFA2 | -5.6098 | 0.046254 |
| THBS3 | 4.750857 | 0.046259 |
| RPL7 | -9.32727 | 0.046285 |
| DNA2 | 4.507027 | 0.046667 |
| OTC | -6.40857 | 0.047085 |
| FOXI1 | -3.21171 | 0.047098 |
| TOP3A | 3.094817 | 0.0471 |
| ATP1A1 | 8.020109 | 0.047134 |
| AES | -6.08899 | 0.047176 |
| ZNF165 | 4.938134 | 0.047176 |
| EXOC5 | 4.317373 | 0.047314 |
| RNASE2 | 3.909809 | 0.047379 |
| PTPRB | -4.02133 | 0.047599 |
| IFNGR2 | 7.184681 | 0.047623 |
| FDPS | 7.051738 | 0.047822 |
| HYAL3 | -3.08298 | 0.048026 |
| SOX6 | -3.22869 | 0.048028 |
| ADRA1A | -2.20732 | 0.048063 |
| PSPH | 6.260311 | 0.048063 |
| ARFIP1 | -5.29438 | 0.048187 |
| GCG | -6.52879 | 0.048299 |
| GPR12 | -3.24194 | 0.048299 |
| COL16A1 | 6.597251 | 0.048362 |
| TRIM21 | -5.20264 | 0.048532 |
| ZNF142 | 3.842354 | 0.04866 |
| CALCOCO2 | -4.78761 | 0.048734 |
| RPL32 | -9.6096 | 0.048932 |
| EIF4H | 7.404883 | 0.048932 |
| WASL | -5.63201 | 0.049151 |
| ABCB10 | 5.65047 | 0.049151 |
| THBD | -5.23827 | 0.049278 |
| CCR5 | 5.902881 | 0.049278 |
| RABEP1 | -3.86721 | 0.049356 |
| PXN | -4.66905 | 0.049367 |
| RPS9 | 8.211477 | 0.049367 |
| LIG3 | 3.767966 | 0.049439 |
| LRRC8B | 3.523495 | 0.049591 |
| PRKAR2B | -5.89285 | 0.049597 |
| SPRR2B | 3.969007 | 0.049636 |
| HK3 | 2.821694 | 0.049652 |
| FCGR3B | 7.58059 | 0.049652 |
| FASN | 5.394194 | 0.049692 |
| SPI1 | 4.169017 | 0.049717 |
| UBE3C | 3.871067 | 0.049727 |
| TNP2 | -1.63165 | 0.049765 |
| ATP6V1A | -6.17097 | 0.049998 |
